# *C*_3_‑Symmetric Photoresponsive Chiral Dopants Based on Tribenzotriquinacene

**DOI:** 10.1021/jacs.5c22128

**Published:** 2026-02-13

**Authors:** Brandon Balamut, Indu Bala, Bahiru P. Benke, Jerry Jose, Michael Mastalerz, Ivan Aprahamian

**Affiliations:** a 6128 Burke Laboratory, Department of Chemistry, 3728 Dartmouth College, Hanover, New Hampshire 03755, United States; b Organisch-Chemisches Institut, Ruprecht-Karls-Universität Heidelberg, Im Neuenheimer Feld 272, Heidelberg 69120, Germany

## Abstract

Doping cholesteric liquid crystals (CLCs) with photoresponsive chiral molecules is an effective strategy for devising responsive soft materials, as it allows for the phototuning of the noncovalent interactions in the CLCs, and hence, their helical pitch and optical properties. Here we describe the use of tribenzotriquinacene-based (TBTQ) hydrazone and azobenzene chiral dopants in the modulation of the helical pitch of the LC host, 5CB. The unique *C*_3_-symmetry of the TBTQ scaffold enhances the noncovalent interactions with the host and thus the chiral information transfer, resulting in helical twisting power (β) values as high as 147 μm^–1^. Notably, the TBTQ hydrazone exhibits an unusual deviation from trends observed so far in previous studies, resulting in larger β values for the *Z* isomer rather than the *E* one. Moreover, the overall β values for the hydrazone-based dopants are unexpectedly higher than those of the azobenzene dopants. These results indicate that the host/guest interactions are better when the photoswitchable chiral dopant is more rigid, as is the case with the H-bonded *Z* state of the hydrazone. The large β values and excellent miscibility of the hydrazone-based dopants in 5CB allowed us to design films that reflect visible structural color. The properties of the azobenzene-based dopants precluded their use in such applications. By codoping 5CB with hydrazone and azobenzene derivatives of opposite chirality, we demonstrated reversible handedness inversion upon photoswitching, providing a versatile soft material platform for reconfigurable photonic materials and colorimetric display technologies.

## Graphical abstract

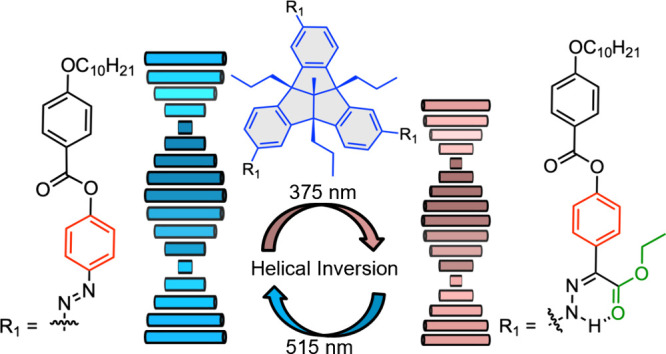

## Introduction

Symmetry provides a powerful structural lever in the design of liquid crystal (LC) dopants,^1^ where the spatial arrangement of substituents can determine the extent of noncovalent interactions with the host LC.^2^ In cholesteric liquid crystals (CLCs), dopant symmetries dictate the extent to which molecular shape and anisotropy control the helical pitch (*P*) of the CLC, giving rise to reflected light and structural color (i.e., λ = *nP*, where λ is the wavelength of reflected light and *n* is the refractive index), and its handedness.^3^ Over the past decades, axially chiral *C*_2_-symmetric dopants, based on binaphthyl,^4^ and more recently triptycene scaffolds,^5^ have emerged as the dominant motifs for amplifying chiral information transfer (i.e., leading to large helical twisting powers (β)) to host LCs. These scaffolds have helped establish fundamental design rules for symmetry-guided chirality transfer in soft matter and photonic materials.^6,7^ The incorporation of molecular photoswitches^8–10^ into such chiral scaffolds allows for the noninvasive and dynamic control over the noncovalent interactions between the dopant and host CLCs, thus allowing for the tuning of the LC’s optical properties^11,12^ and even handedness.^13^ Among the most versatile classes of photoswitches, azobenzenes^14–16^ and hydrazones^17–25^ have proven particularly attractive in such applications,^19–33^ because of their distinct photochromic properties (e.g., high photostationary states and quantum yields (PSS and Φ, respectively),^34,35^ reversible isomerization accompanied by large geometric shape changes, and tunable thermal half-lives (τ_1/2_)^36–39^ ).

In sharp contrast, *C*_3_-symmetric scaffolds (Scheme 1), while well-studied in discotic LCs,^40–42^ are underexplored as CLC dopants,^43,44^ despite their prominent roles in asymmetric catalysis,^45–50^ host–guest interactions, molecular recognition,^51–53^ and functional materials.^54^ Such scaffolds are also known to amplify stereochemical information,^55^ promote cooperative interactions, and bias assembly pathways toward long-range chiral order,^56^ making them ideal for LC applications. Based on these properties, we speculated that a *C*_3_-framework such as tribenzotriquinacene (TBTQ),^57–68^ when combined with photoswitchable units, might offer a unique opportunity to study how photoreversible *C*_3_-symmetric chiral dopants can be used in controlling the self-assembly of CLCs.

**Scheme 1:**
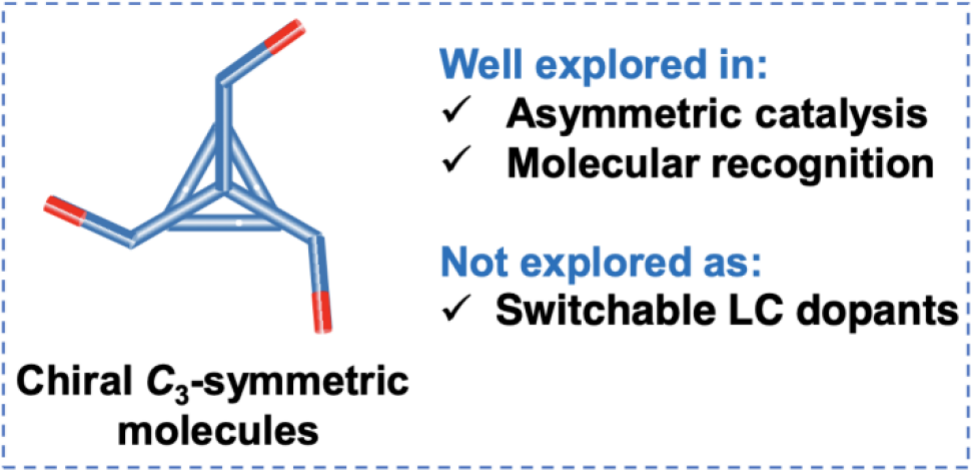
Chiral *C*_3_-Symmetric Framework and Potential Uses

Recently, we have shown how using a chiral dopant attached to two hydrazone switches with distinct photoswitching properties can result in unusual phase changes in LCs.^27^ This result prompted us to speculate about how the mixing of two chiral dopants with different types of photoswitches might affect LC properties. The use of different photoswitches in the same application is nontrivial in general, with few successful examples reported in solution,^69–71^ and the solid-state,^72–75^ but far less so in LCs.^76,77^ A variety of encumbering limitations (e.g., spectral overlap and undesired energy transfer between the chromophores) are the culprit behind this difficulty, which is compounded in LCs, where different types of dopants can result in phase separation. Hydrazones and azobenzenes exhibit overlapping absorption bands in the UV-region but display distinct well-separated bands in the visible range. Moreover, their τ_1/2_ values differ substantially. Based on these differences, we hypothesized that the selective photoisomerization and independent thermal reversion of each photochrome will provide us with unique handles to couple and average the individual β value contributions of each dopant to the overall helical chirality of the LC.

Here we report on the development of *C*_3_-symmetric TBTQ-based hydrazone ((−)-*P*/(+)-*M* (**1**)) and azobenzene ((−)-*P*/(+)-*M* (**2**)) photoswitchable chiral LC dopants (Scheme 2). Surprisingly, the β values of the hydrazone-based dopants do not follow the trend reported so far for such switches (i.e., *Z*→*E* photoisomerization results in a smaller β value),^19^ whereas the azobenzene-based dopants unexpectedly result in overall β values that are lower than their hydrazone counterparts.^16^ We speculate that the additional third switchable arm in the *C*_3_ dopants results in these unexpected outcomes. We next used the hydrazone dopants to effectively manipulate the light reflection to generate colored films that reflect visible light. The azobenzene dopants on the other hand resulted in an isotropic phase or alignment issues in the appropriate LC films, and hence, could not be used in selective color reflection. Finally, by taking advantage of the differences in the photoswitching properties and β values of the hydrazone and azobenzene-based dopants, and by mixing different ratios of dopants with opposite handedness, which is a unique strategy, we were able to show orthogonal control and photoinduced helical inversion of the CLC assemblies.

**Scheme 2:**
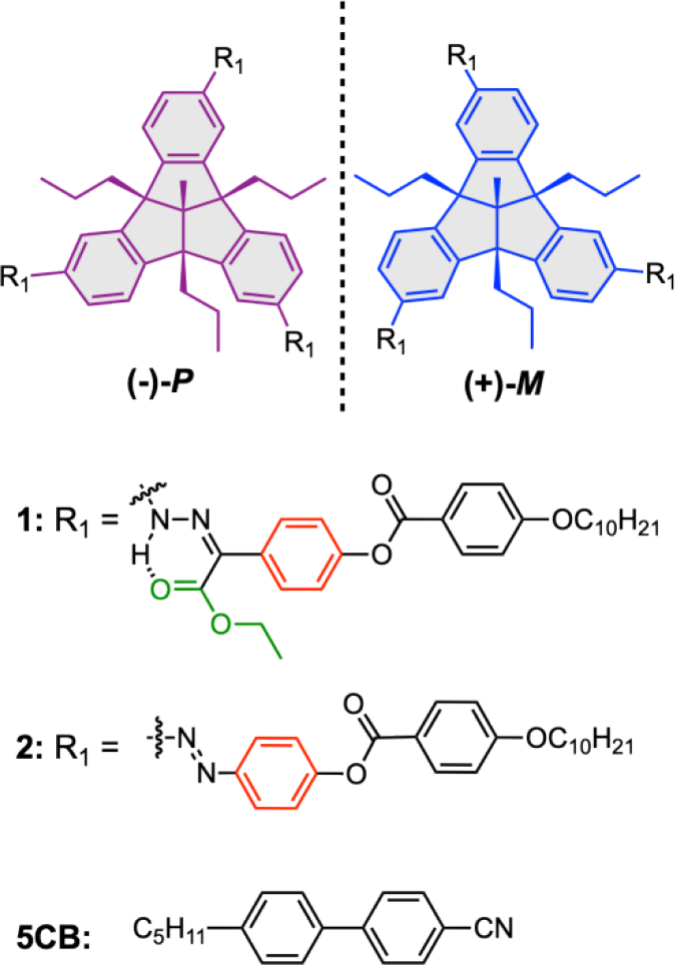
Chemical Structures of the Enantiomers of TBTQ-Based Hydrazone and Azobenzene Photoswitches and the Achiral Nematic LC, 5CB

## Results

The chiral photochromic switches **1** and **2** consist of a central TBTQ motif connected at the phenylene units to 4-decyloxybenzoate functionalized hydrazone and azo photochromic units, respectively (Scheme 2). The alkoxy chains were chosen to promote miscibility and improve dispersion interactions with the achiral nematic LC host, 5CB, amplify the geometric change upon photoisomerization, and enable comparison with previously studied sytems.^16,19^ The synthesis^58,62^ and characterization of the (−)-*P*/(+)-*M* dopants are described in the Supporting Information (Scheme S1 and Figures S1–S10). The *ZZZ* and *EEE* isomers of **1** and **2**, respectively, were isolated, after column chromatography, as the major configurational isomers. The photophysical and photoisomerization properties of the dopants were studied using UV–vis, ^1^H NMR and circular dichroism (CD) spectroscopies, and their identities confirmed using high-resolution mass spectrometry (Figure 1 and Figures S1–S29).

**Figure 1:**
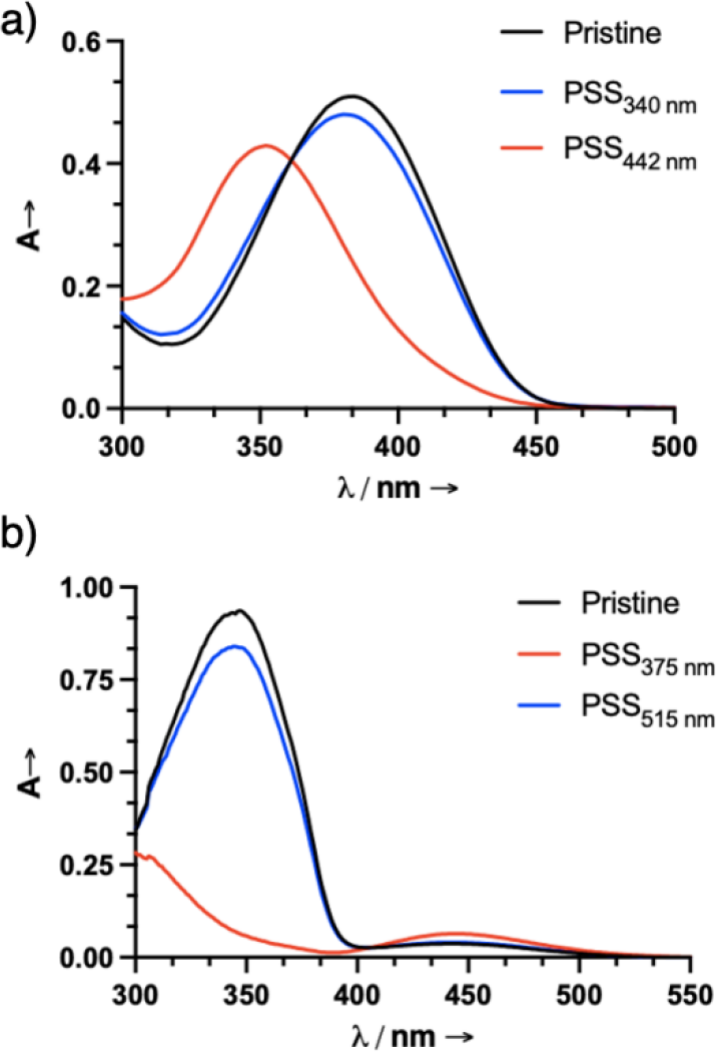
UV–vis absorption spectra of **1** and **2** (1.0 × 10^–5^ M) in toluene. a) Irradiation of **1** with 442 nm light results in *Z*→*E* isomerization, and the process can be reversed with subsequent irradiation with 340 nm light. b) Irradiation of **2** with 375 nm light results in *E*→*Z* isomerization, and the process can be reversed with subsequent irradiation with 515 nm light.

Irradiation of **1**-*ZZZ* (maximum absorption (λ_max_) at 384 nm, absorption coefficient (ε) = 87,700 M^–1^cm^–1^) in toluene with 442 nm light results in a hypsochromic shift (λ_max_ = 374 nm, ε = 76,400 M^–1^cm^–1^) and affords a PSS_442_ of 91% *EEE* (shortened as *E* moving forward). This process can be reversed by irradiation with 340 nm light, yielding 81% of the *ZZZ* (shortened as *Z* moving forward) form at PSS_340_. Interestingly, less than 5% of the *ZZE* and *EEZ* isomers were formed during the photoirradiation process (Figures S13 and S14), indicating that the hydrazones switch independently and efficiently. The UV–vis spectrum of **2**-*E* displays the characteristic dual absorption band of azobenzenes, with the more prominent band (λ_max_ = 347 nm, ε = 93,600 M^–1^ cm^–1^) stemming from the π–π* transition and the weaker one (λ_max_ = 443 nm, ε = 6,400 M^–1^ cm^–1^) from the n−π* transition. Irradiation of a pristine sample of **2** in toluene with 375 nm light affords a PSS_375_ of 87% *Z*, with very little formation (i.e., less than 5%) of the intermediate isomers, and is accompanied by a decrease in the π–π* band intensity and a slight increase of the n−π* band. The isomerization process can be reversed by irradiation with 515 nm light, yielding a PSS_515_ comprising 79% of the *E* state, and 20% contribution from the *ZZE* and *EEZ* isomers (Figures S17 and S18). The *Z*→*E* isomerization of **1** and its reverse process have a quantum yield (Φ) of 2.2 ± 0.1% and 1.8 ± 0.1%, respectively (Table S1 and Figures S19–S22), whereas the observed Φ for the *E*→*Z* isomerization of **2** and its reverse process were measured to be 11.6 ± 1.3% and 23.0 ± 0.9%, respectively (Table S2 and Figures S23–S26). The τ_1/2_ for **1** and **2** were calculated to be 4.5 ± 0.6 years and 5.6 ± 0.4 days (Table S3 and Figures S30 and S31), respectively, showcasing the expected thermal stability of the hydrazone-based dopant in comparison to the azobenzene-based one. Multiple switching cycles were performed with **1** and **2**, and while some minimal photofatigue was observed for the former, the latter showed no changes in absorption intensities after consecutive switching cycles (Figures S12 and S16, respectively).

To study the effect of the TBTQ dopants on the LC properties of achiral nematic hosts, **1** and **2** were doped into 5CB, yielding cholesteric phases. The dopants showed excellent solubility in 5CB and induced opposite handedness depending on which enantiomer was used (i.e., (−)-*P* or (+)-*M*). The β values of the dopants were measured using the Grandjean-Cano wedge method^78^ before and after irradiation with 442/340 and 375/515 nm light for **1** and **2** respectively (Figure 2, Table S4, and Figures S32–S39). As expected, the β values of the (−)-*P* or (+)-*M* enantiomers were the same. As an example, we will focus here on pristine (+)-*M*-**1**-*Z*, which has significant dopant-host interactions as evidenced by its large β value of +147 μm^–1^. However, and unlike most other hydrazone-based dopants studied so far,^19–27^ upon photoisomerization to the *E* form a decrease in the β value of 19% to +119 μm^–1^ is observed, indicating that the interactions between the host and guest are weaker. Irradiating the *E* rich state with 340 nm light, surprisingly results in almost complete restoration of the β value to +145 μm^–1^ although at the PSS the *Z* isomer ratio is almost 20% less than in the pristine state. Pristine (+)-*M*-**2**-*E* has a β value of +93 μm^–1^, indicating weaker interactions with 5CB than **1**, which is contrary to what we found in the triptycene-based dopants.^16^ Irradiation with 375 nm light to the *Z* rich state induces a sharp reduction in the β value, accompanied by unwinding of the helical assembly and loss of the CLC texture. Such disruptions are common in azobenzene-based CLCs^79^ and preclude precise quantification of the β value, which we estimate to be less than 10 μm^–1^. Irradiation with 515 nm light, results in a β value of +51 μm^–1^, which as expected is lower than the pristine value because of the PSS isomer ratio. Heating the sample at 100 °C for 120 s results in thermal equilibration, and the value reverts to +93 μm^–1^.

**Figure 2:**
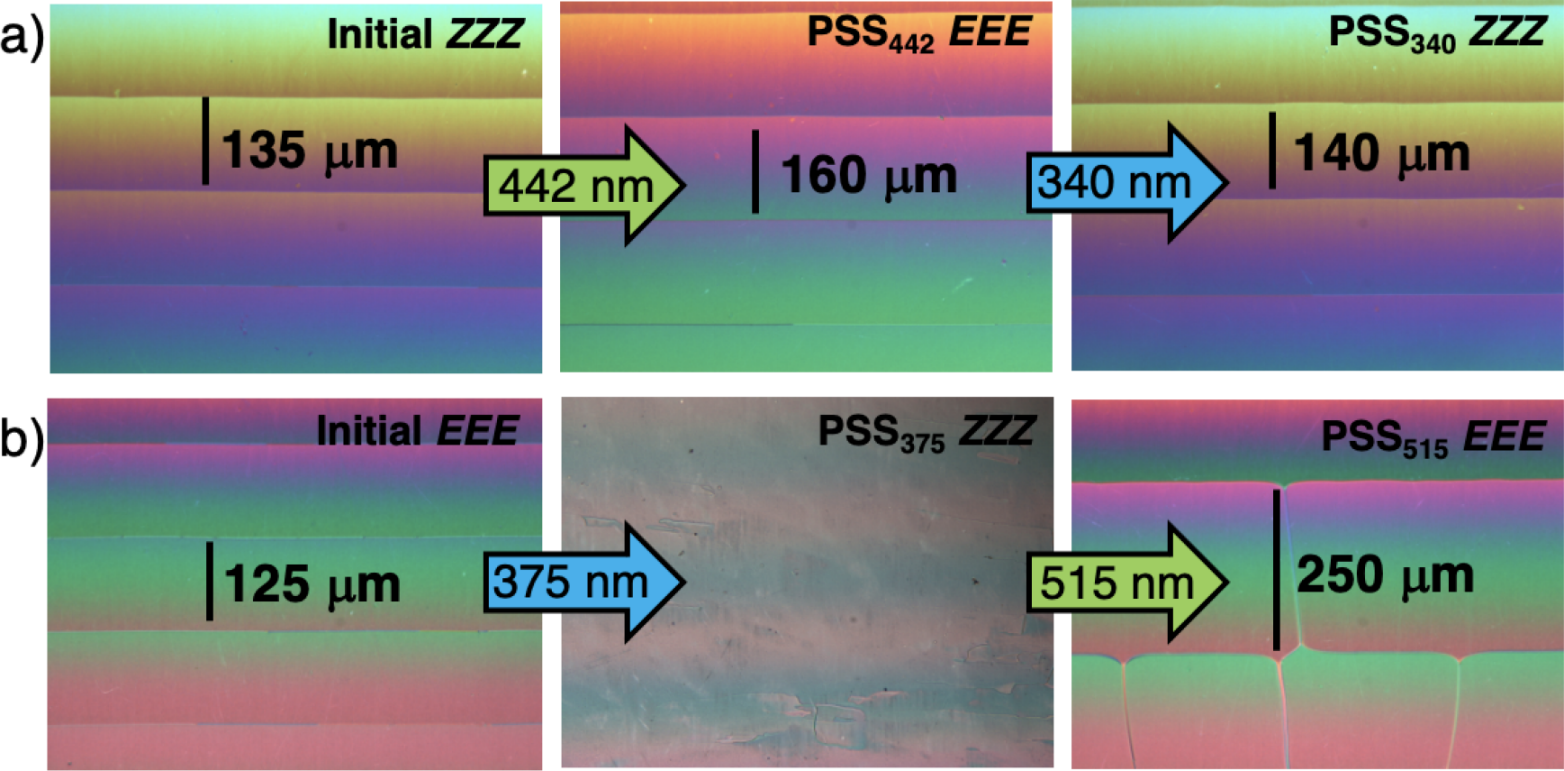
Polarized optical micrographs of a) **1** (0.20 mol %) and b) **2** (0.20 mol %) after irradiation with 442/340 and 375/515 nm light in KCRK05 and KCRK07 wedge cells, respectively. The measured distances of the Cano lines (units: μm) were used to calculate the helical pitch of the CLC and the *β* values before and after photoisomerization.

## Discussion

These results show that the rigid, bowl-like *C*_3_ structure of TBTQ allows for enhanced chirality transfer in both types of dopants. The β value of +147 μm^–1^ is to date the highest one we have measured for a hydrazone-based system. Uniquely though, it is the more rigid H-bonded *Z* isomer of **1**, and not the conformational flexible *E* form that results in the higher β value. This result tells us that in this *C*_3_ structure, the rigidity of the photoswitch is an important factor in determining the chiral information transfer. This point is further validated by the fact that the β values of the *E* state of **1** and **2** are similar, indicating that the nature of the flexible arms is less important than the overall shape of the TBTQ unit in the chiral information transfer.

To take advantage of the large β values of the dopants, reflective adaptive films of **1** or **2** (>3.0 mol %) were prepared using 5CB as the host (Figure 3). Notably, the enhanced thermal stability of **1** allowed us to lock in different PSSs, and in turn, helical pitch lengths and thus reflected visible colors (Figure 3a and Figures S40 and S41). The transmittance data obtained from **1**, measured as a function of irradiation wavelength, showed excellent control over the reflection color from the visible to the NIR region (e.g., 540 to 750 nm for 2.7 mol %, and <400 to 610 nm for 3.4 mol % doping) allowing us to draw different shapes and colors on the LC canvas (Figure 3b). Repeated write/erase cycles using the same film showed no evidence of photofatigue. Reflective films of **2** on the other hand, could not be generated because of a cholesteric to isotropic phase change at concentrations above 1.4 mol %. At lower concentrations, the *E* rich state (i.e., pristine and PSS_515_) did not yield well aligned helical structures resulting in the scattering of light. However, upon irradiation with 375 nm light, a well aligned cholesteric phase was obtained upon elongation of the helical pitch, resulting in full transmission of incident light (Figure 3c), though visible light reflection was not observed because of the low β value.

**Figure 3:**
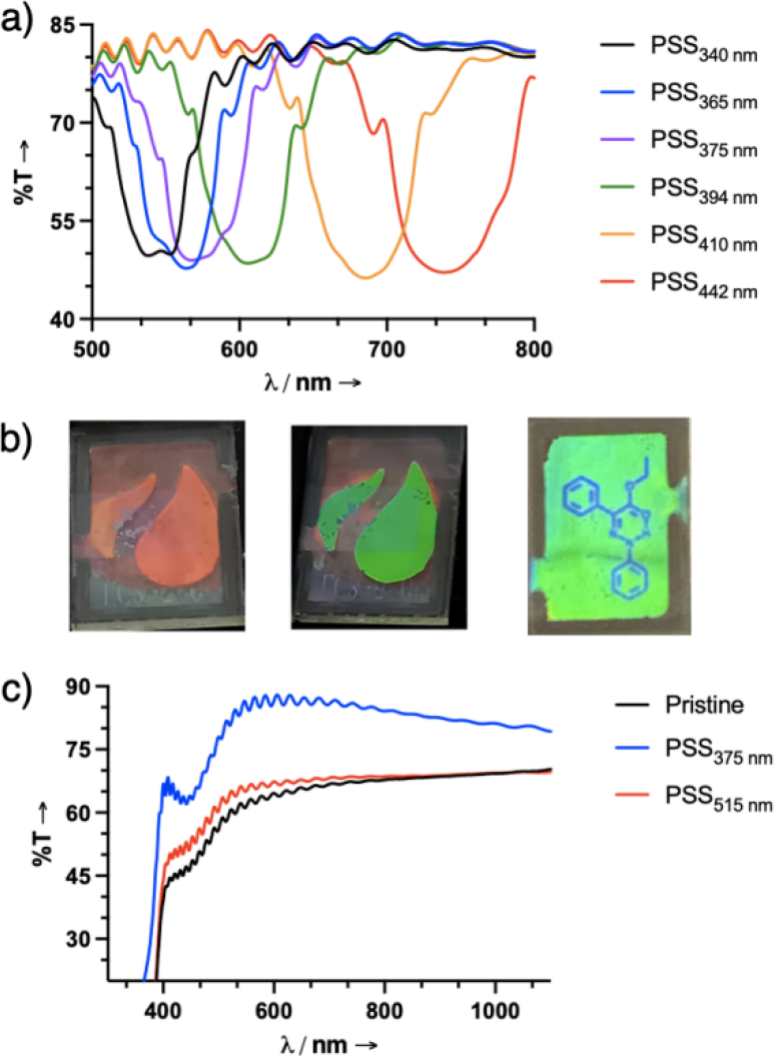
a) Modulation of the structural color of 5CB with **1** (2.7 mol %) in a LC 3–5 cell as a function of the irradiation wavelength. b) Photomicrographs of **1** (2.7 and 3.4 mol %) in a LC 3–5 cell (5 μm gap, planar cell) were generated by irradiation through grayscale masks in the shape of a flame or a phenyl hydrazone photoswitch with 442 nm light, showing how different shapes in red, green and blue colors can be reflected from the LC surface. c) Reflective adaptive films of **2** (1.4 mol %) resulted in scattering of light and could revert to an aligned CLC texture upon irradiation with 375 nm light.

Next, mixtures of opposite handed enantiomers of **1** and **2** were prepared to study their effect on the properties of the LC (Figure 4 and Figures S42 and S43). We hypothesized that by modifying the ratio of (−)-*P*-**1** and (+)-*M*-**2**, we could take advantage of the large β values of **1** and the low β value of the *Z* rich state of **2** to induce handedness inversion. The opposite enantiomeric pair, (+)-*M*-**1** and (−)-*P*-**2**, would give similar behavior if they were used. First we studied the photoswitching of mixtures of the two dopants both in solution (Figures S44 and S55) and in the LC (Figure 4) and confirmed that they do not alter each other’s photophysical and photoswitching properties, and that they act independently, allowing for their orthogonal control using light irradiation (i.e., **1** does not absorb 515 nm light, whereas **2** does). Next we measured the β value of a mixture of (−)-*P*-**1** and (+)-*M*-**2** (30:70 ratio, respectively, 0.23 mol %) and found it to be +22 μm^–1^ (Figure S42), which matches the calculated value (i.e., 0.3(−145) + 0.7­(+93) = +21.6 μm^–1^). Upon irradiation with 375 nm light, the CLC phase underwent helical inversion and resulted in a β value of – 15 μm^–1^ (Figure 4a). Subsequent irradiation with 515 nm reverted the system to the starting helicity, with a β value estimated < + 5 μm^–1^ (Figure S42), a value dictated by the PSS of the azobenzene dopant. The mixture was also loaded into a homeotropically aligned cell and inversion was observed upon irradiation with 375 and 515 nm light (see Movies S1 and S2, respectively). Cholesteric contact experiments with left-handed cholesterol oleyl carbonate (Figure S56) confirm that a left- to right-handed inversion of helicity occurs upon irradiation with 375 nm, whereas irradiation with 515 nm light reversibly restores the starting left-handed helicity, as evidenced by the emergence and disappearance of discontinuous regions at the interface, respectively. Increasing the concentrations of **1** and **2** (>1.0 mol %) while keeping the same ratio, resulted in isotropic films instead of the targeted visible light reflecting LC surfaces. We speculate that since the amount of **2** is more than 2-fold than that of **1**, the phase disruption is occurring because of the incompatibility of the azobenzene-based dopant with the LC. To serve as a control, mixtures of similar enantiomers of (+)-*M*-**1** and (+)-*M*-**2** (32:68 ratio, respectively, 0.12 mol %) were also prepared. The β values were similar to the average contributions of each individual dopant ranging from +113 μm^–1^ to +61 μm^–1^ (Figure S43) and as expected, helical inversion was not observed (Figure S57). These results demonstrate that this novel method of judicious mixing of dopants with opposite handedness and appropriate β values is a viable strategy for controlling helicity inversion in LCs.

**Figure 4:**
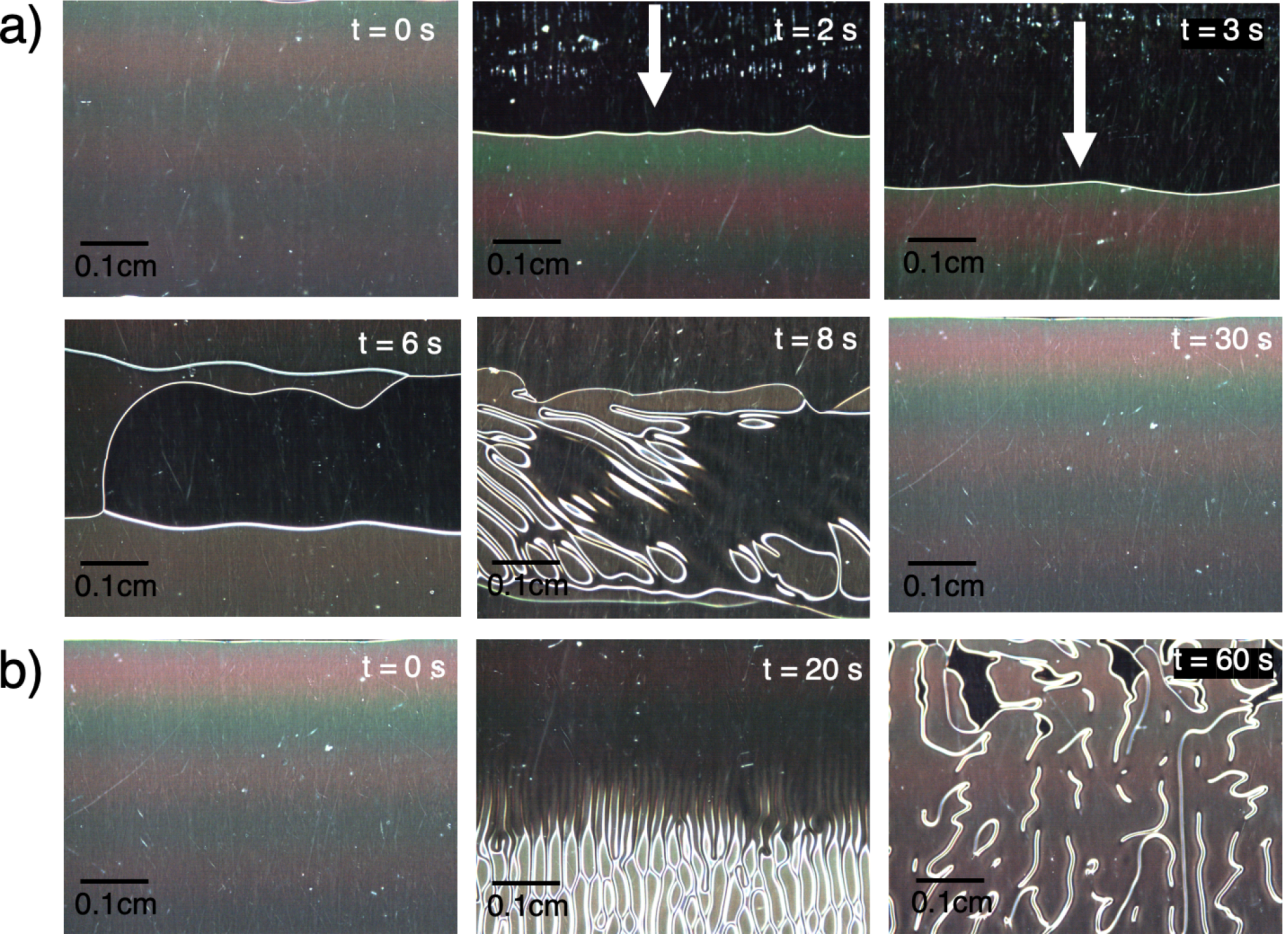
Modulation of the helical pitch of (−)-*P*-**1** and (+)-*M*-**2** in 5CB (30:70 ratio, respectively, 0.23 mol %) in a KCRK05 cell. a) After 2 s of irradiation with 375 nm light, the Cano lines begin to disappear from the edge of the wedge cell, as denoted by the white arrows. Within 6 s after their complete disappearance, the lines reform, as indicated by the oily streak texture, and are properly aligned with the helicity inverted, after 30 s of irradiation. b) Within 20 s of exposure to 515 nm light the Cano lines disappear, followed by the reemergence of the cholesteric phase, which reverts to the starting handedness within 60 s.

## Conclusion

In summary, we demonstrated the use of a new *C*_3_-symmetric chiral scaffold, TBTQ, by incorporating it into hydrazone- and azobenzene-based dopants. There are clear advantages to using the hydrazone-based systems as they result in large β values, excellent compatibility with the LC host, modulation of long-lived reflective visible colors, and repeated write/erase cycles. On the other hand, the azobenzene-based system results in phase separation and no reflective surfaces. We speculate that one of the reasons behind this disparity is that in such a *C*_3_-symmetric chiral scaffold the rigidity of the switch is critical for imparting large β values. This line of reasoning explains why the *Z* isomer of the hydrazone has a higher β than the *E* one, which is contrary to all other reported dopants based on this photoswitch.^5,25–27^ We have also shown that the straightforward mixing dopants with varying β values and opposite helicities allows for the photoinduced helical inversion of the CLC. While a few reports in the literature,^76,77,80^ describe the mixing of different chiral switches for the enhanced and orthogonal control over LC properties, this is the first instance where mixing is used for handedness inversion. Overall, the work showcases the promise of using TBTQ as a new chiral scaffold, in part because of the large chiral induction with 5CB; moreover, it emphasizes how the nature of the photoswitch can have a large impact on the properties of the chiral photoswitchable dopant.

## Supplementary Material







## Notes

General methods, experimental procedures, NMR spectra of key compounds, photoisomerization studies, kinetic studies, and details about doping experiments including movies (PDF)
*C*_3_ 375 nm irradiation (MP4)
*C*_3_ 515 nm Irradiation (MP4)
