# Supplementary material for: C 3‑Symmetric Photoresponsive Chiral Dopants Based on Tribenzotriquinacene
Source: J Am Chem Soc. 2026 Feb 13;148(7):7887–94. doi: 10.1021/jacs.5c22128 (PMC12951436; doi:10.1021/jacs.5c22128)
Supplement: Supplementary file 1 [file ja5c22128_si_001.pdf]

Supporting Information

C_3-Symmetric Photoresponsive Chiral Dopants Based on Tribenzotriquinacene

Brandon Balamut[a], Indu Bala[a], Bahiru P. Benke[b], Jerry Jose,[a] Michael Mastalerz[b], and Ivan Aprahamian[a]*

[a]Department of Chemistry, Dartmouth College, Hanover, New Hampshire, 03784, USA
[b]Organisch-Chemisches Institut, Ruprecht-Karls Universität Heidelberg, 69120 Heidelberg, Germany

Email: ivan.aprahamian@dartmouth.edu

Table of Contents

1. **General Methods** **S3**
2. **Synthesis and Characterization of Compounds** **S4**
3. **NMR Characterization** **S7**
4. **Photoisomerization Studies** **S12**
5. **Quantum Yield Determination** **S17**
6. **Circular Dichroism Measurements** **S23**
7. **Thermal Isomerization Half-Life Determination** **S25**
8. **HTP and Reflectance of Adaptive Films** **S28**
9. **Co-Doping Hydrazone and Azobenzene in Solution and Liquid Crystal** **S34**
10. **References** **S42**

1. General Methods

All reagents and starting materials were obtained from commercial sources and used without further purification unless otherwise noted. All reactions were done under normal atmosphere unless otherwise noted. Compounds were purified by column chromatography using silica gel (SiliCycle®, 60 Å, 230-400 mesh) as the stationary phase and eluting solvents are reported as ratios unless otherwise noted. Recrystallizations were performed with HPLC grade solvents. Deuterated solvents were obtained from Cambridge Isotope Labs and used without further purification. ^{1}H and ^{13}C NMR spectra were recorded on 500 or 600 MHz instruments with working frequencies of 500.13 and 600.13 MHz for ^{1}H nuclei and 125.8 or 150.9 MHz for ^{13}C nuclei, respectively. Chemical shifts are quoted in ppm relative to tetramethylsilane (TMS), using the residual solvent peak as the reference standard. ESI mass spectra were obtained on a Shimadzu LCMS-8030 mass spectrometer. UV-Vis and transmittance spectra were recorded on a Shimadzu UV-1800 UV-Vis spectrophotometer.

Irradiation experiments were conducted with a stand-alone xenon arc lamp system (Model:LB-LS/30, Sutter Instrument Co.), outfitted with a SMART SHUTTER controller (Model: LB10-B/IQ, Sutter Instrument Co.) and a liquid light guide LLG/250. 340 (part number: 340HC10-25), 365 (365HC10-25), 375 (375HC10-25), 394 (part number: 394HC10-25), 410 (part number: 410FS10-25), 442 (part number: 442FSX10-25), and 515 (part number: 515FSX10-25) nm light filters, purchased from Andover Corporation, were used in the irradiation experiments.

The textures of the liquid crystal samples were evaluated using an Olympus BX53 polarized optical microscope, the photomicrographs were captured using an INSTEC MITO2-MC camera. The planar and homeotropically aligned liquid crystal cells were purchased from INSTEC, and the wedge cells were purchased from EHC Co Ltd. Liquid crystalline host 5CB was purchased from Ambeed.

2. Synthesis and Characterization of Compounds

Scheme S1. Synthesis of chiral hydrazone-based C_3-symmetric tribenzotriquinacene.

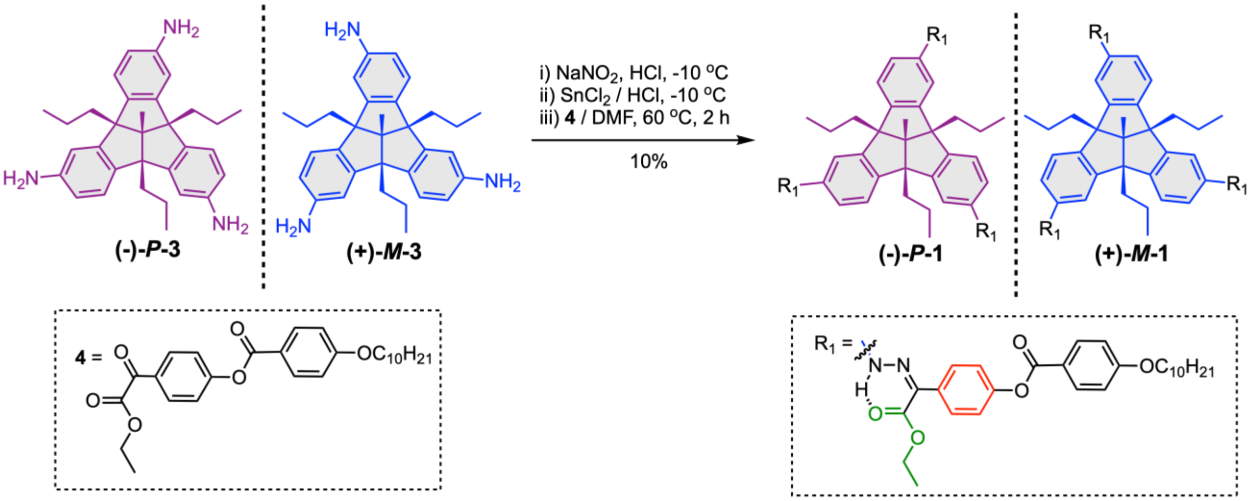

(-)-*P*-3/(+)-*M*-**3**: These enantiomers were synthesized and separated by using a reported procedure [S1] and their identity confirmed by comparing the obtained ^{1}H NMR spectrum with the reported one; ^{1}H NMR (500 MHz, CD_3CN) δ 6.97 (d, J = 8.2 Hz, 3H), 6.55 (d, J = 2.2 Hz, 3H), 6.43 (dd, J = 8.2, 2.2 Hz, 3H), 2.04 – 1.96 (m, 6H), 1.50 (s, 3H), 1.14 (m, 6H), 0.89 (t, J = 7.4 Hz, 9H).

4: This compound was synthesized using a reported procedure[S2] and its identity confirmed by comparing the obtained ^{1}H NMR spectrum with the reported one; ^{1}H NMR (500 MHz, $CDCl_3$) δ 8.36 – 8.31 (m, 2H), 8.21 – 8.12 (m, 4H), 7.34 – 7.29 (m, 2H), 4.49 (q, J = 7.1 Hz, 2H), 4.33 (t, J = 6.7 Hz, 2H), 1.77 (p, J = 6.8 Hz, 2H), 1.49 – 1.41 (m, 4H), 1.40 – 1.21 (m, 12H), 0.92 – 0.85 (m, 3H).

(-)-*P*-**1**/(+)-*M*-**1**: A precooled solution of sodium nitrite (26 mg, 0.38 mmol) in 0.5 mL water was added dropwise over 45 min to a solution of (-)-*P*-**3** or (+)-*M*-**3** (50 mg, 0.11 mmol) in 2 mL of water and 1 mL of hydrochloric acid at -10 °C. The mixture was stirred for 20 min and then a precooled tin(II)chloride suspension (183 mg, 0.97 mmol) in 2 mL of hydrochloric acid was added dropwise. The reaction was allowed to proceed at -10 °C for 15 min and then **4** (161 mg, 0.35 mmol) in 5 mL DMF was added in one portion. The mixture was heated to 60 °C under nitrogen atmosphere for 2.5 h. After cooling to room temperature, the mixture was neutralized with sodium bicarbonate, diluted with water, and extracted with methylene chloride. The combined organic

fractions were washed twice with water and once with brine. The organic layer was dried under vacuum and the mixture was subjected to column chromatography (8:2 Hexane/Methylene Chloride), yielding 40 mg of compound (-)-*P*-**1** (10% yield) and 40 mg of compound (+)-*M*-**1** (10% yield) as yellow solids; ^{1}H NMR (600 MHz, CD_2Cl_2) δ 12.47 (s, 3H), 8.14 (d, J = 8.7 Hz, 6H), 7.71 (d, J = 8.5 Hz, 6H), 7.29 (d, J = 8.9 Hz, 3H), 7.21 (d, J = 8.5 Hz, 6H), 7.16 (d, J = 5.8 Hz, 6H), 6.99 (d, J = 8.7 Hz, 6H), 4.36 (q, J = 7.1 Hz, 6H), 4.05 (t, J = 6.5 Hz, 6H), 2.21 – 2.11 (m, 6H), 1.85 – 1.79 (m, 6H), 1.51 – 1.45 (m, 6H), 1.36 (t, J = 7.1 Hz, 12H), 1.26 (m, 42H), 0.94 (t, J = 7.2 Hz, 9H), 0.89 (t, J = 6.8 Hz, 9H) ppm. ^{13}C NMR (151 MHz, CD_2Cl_2) δ 165.46, 164.20, 151.08, 150.35, 143.49, 143.02, 134.95, 132.72, 130.26, 127.19, 125.19, 122.10, 121.81, 114.86, 114.75, 110.33, 69.01, 67.64, 61.69, 41.55, 32.48, 30.28, 30.14, 29.91, 29.68, 26.52, 23.26, 21.03, 15.40, 14.55, 14.45 ppm. m/z found [M-H$^+$] for $C_{113}H_{139}N_6O_{15}^+$ 1820.0231 (calcd. 1820.0298).

Scheme S2. Synthesis of chiral azobenzene-based C_3-symmetric tribenzotriquinacene.

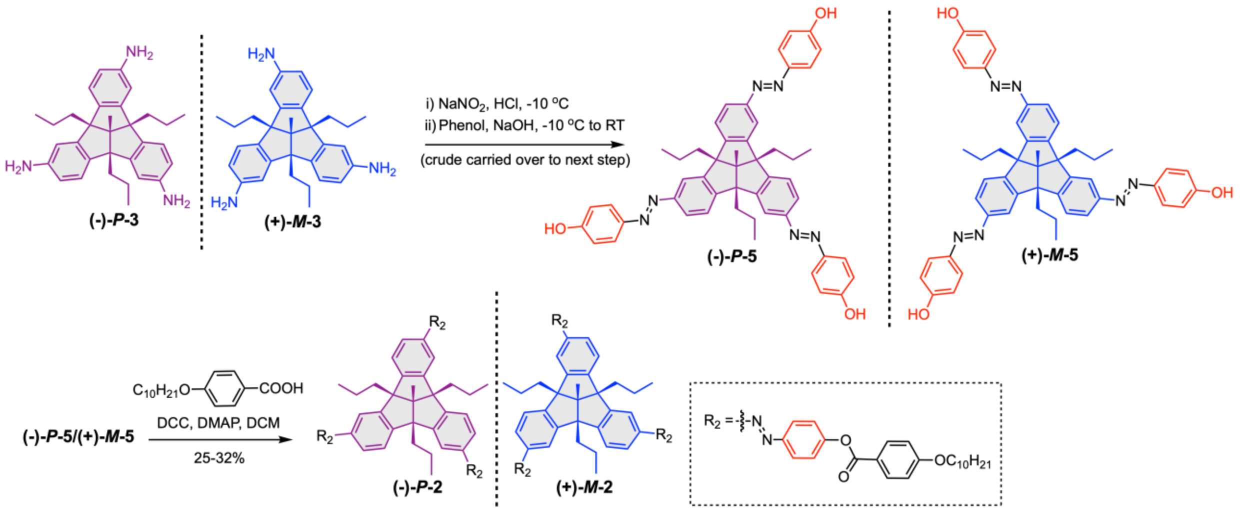

(-)-*P*-**5**/(+)-*M*-**5**: A precooled solution of sodium nitrite (30 mg, 0.37 mmol) in 0.5 mL water was added dropwise over 45 minutes to a solution of (-)-*P*-**3** or (+)-*M*-**3** (0.05 g, 0.11 mmol) in 2 mL of water and 1 mL of hydrochloric acid at -10 °C A solution of phenol (0.034 g, 0.37 mmol) and sodium hydroxide was then added dropwise into the mixture and the reaction temperature was gradually brought up to room temperature and mixed for an additional 45 minutes. The crude mixture was extracted with ethyl acetate and washed twice with water and once with brine and dried under vacuum. The crude products (-)-*P*-**5** and (+)-*M*-**5** were carried over to the next step without further purification.

(-)-*P*-**2**/(+)-*M*-**2**: A solution of 4-(decyloxy)benzoic acid (0.026 g, 0.07 mmol), DCC (0.025 g, 0.12 mmol), and DMAP (0.001 g, 0.01 mmol) were mixed in 5 mL of dry methylene chloride at 0 °C. After 20 minutes, compound (-)-*P*-**5**/(+)-*M*-**5** (0.04 g, 0.05 mmol) was added and the solution stirred for 12 hours. The crude mixture was extracted with methylene chloride, washed twice with water and once with brine, and dried under rotary vacuum. The mixture was subjected to column chromatography (1:9 EtOAc:Hexane) yielding 26 mg of compound (-)-*P*-**2** (32% yield) and 20 mg of compound (+)-*M*-**2** (25% yield) as yellow solids; ^{1}H NMR (500 MHz, CD_2Cl_2) δ 8.16 – 8.12 (m, 6H), 8.02 – 7.98 (m, 6H), 7.97 (d, J = 1.8 Hz, 3H), 7.81 (dd, J = 8.3, 1.8 Hz, 3H), 7.62 (d, J = 8.3 Hz, 3H), 7.40 – 7.34 (m, 6H), 7.03 – 6.97 (m, 6H), 4.06 (t, J = 6.6 Hz, 6H), 2.41 – 2.27 (m, 5H), 1.86 – 1.78 (m, 6H), 1.51 – 1.45 (m, 6H), 1.42 – 1.20 (m, 47H), 1.00 (t, J = 7.3 Hz, 8H), 0.91 – 0.86 (m, 9H); ^{13}C NMR (151 MHz, CD_2Cl_2) δ 191.90, 164.94, 164.20, 153.56, 153.20, 151.60, 150.67, 149.34, 132.62, 124.71, 124.27, 122.91, 122.75, 121.64, 118.91, 114.77, 68.88, 67.69, 43.20, 41.01, 32.31, 29.97, 29.96, 29.76, 29.73, 29.49, 26.35, 23.09, 20.93, 15.23, 14.28; ESI-MS: *m/z* found [M–H^+] for $C_{101}H_{121}N_6O_9^+$: 1561.9190 (calcd. 1562.9140).

3. NMR Characterization

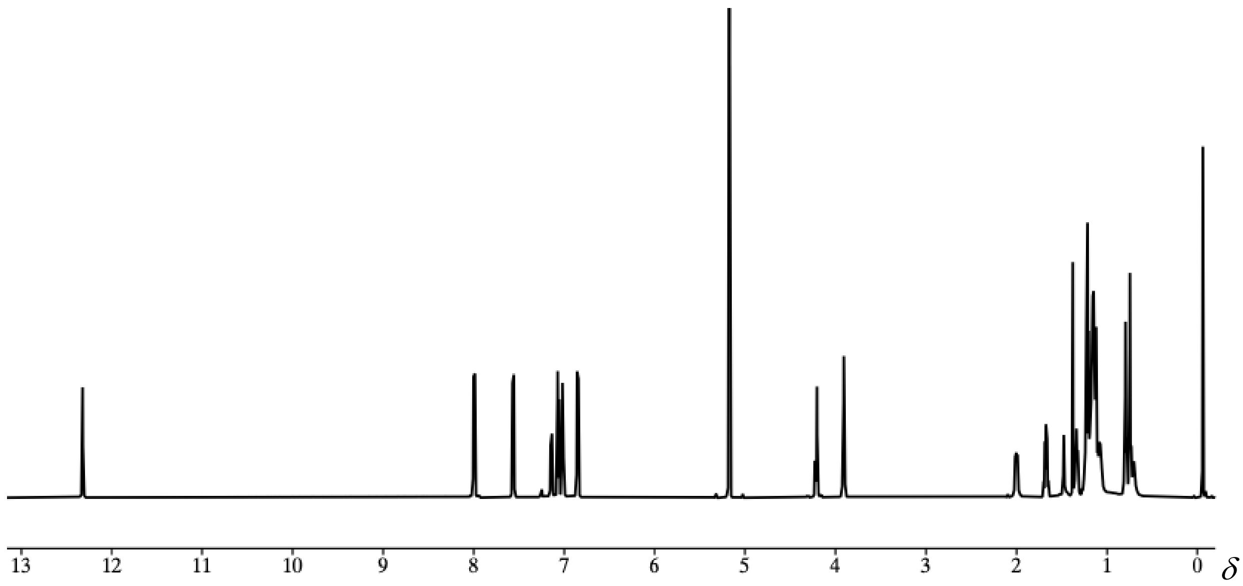

Figure S1. ^{1}H NMR spectrum of (+)-(*M*)-**1** in CD_2Cl_2 at 294 K.

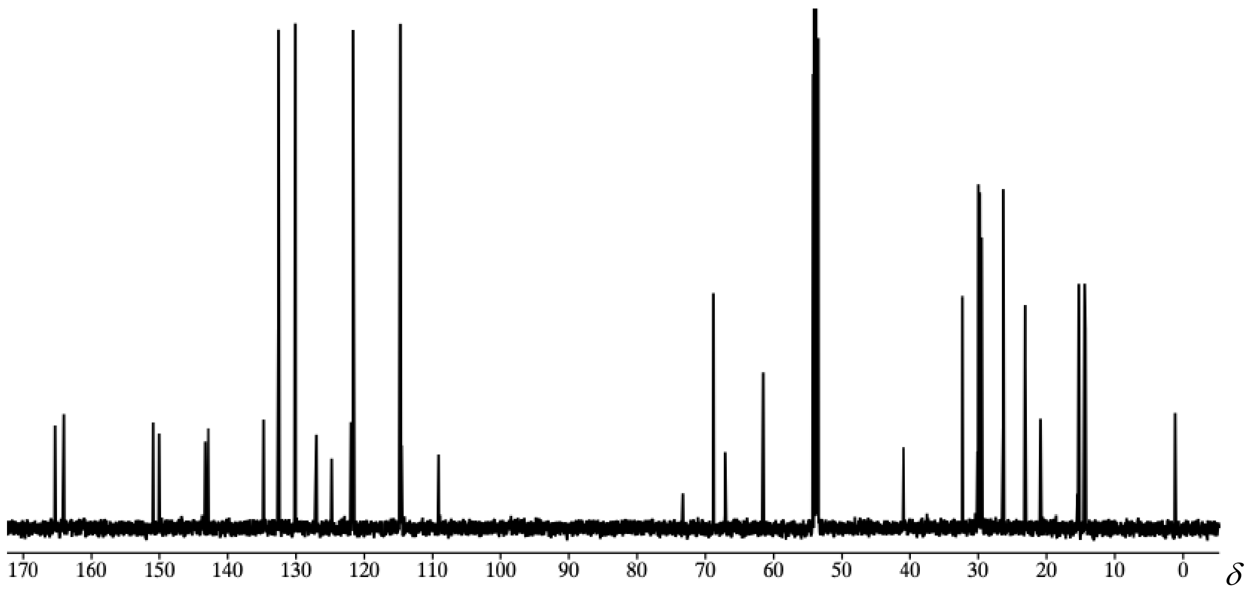

Figure S2. ^{13}C NMR spectrum of (+)-(*M*)-**1** in CD_2Cl_2 at 294 K.

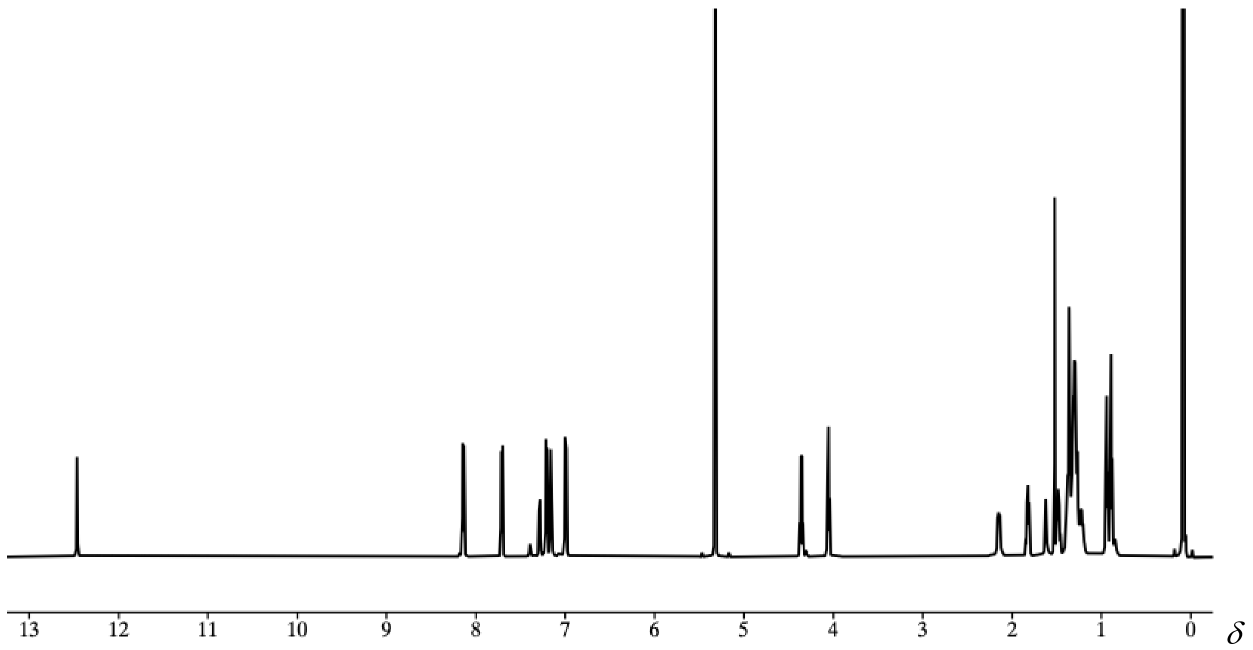

Figure S3. ^{1}H NMR spectrum of (-)-(*P*)-**1** in CD_2Cl_2 at 294 K.

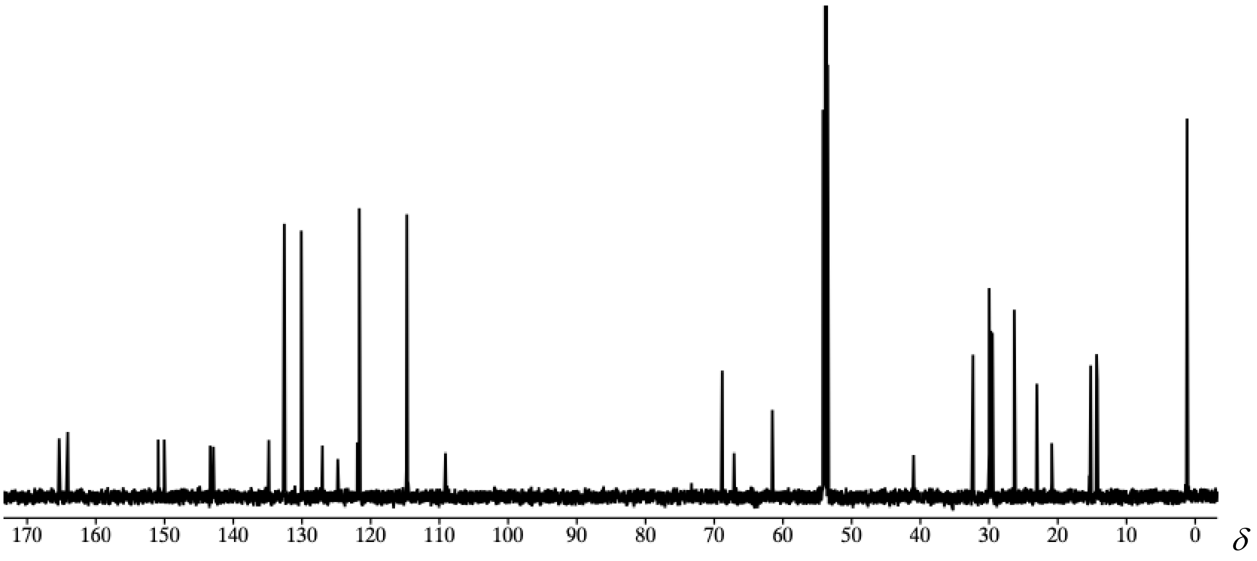

Figure S4. ^{13}C NMR spectrum of (-)-(*P*)-**1** in CD_2Cl_2 at 294 K.

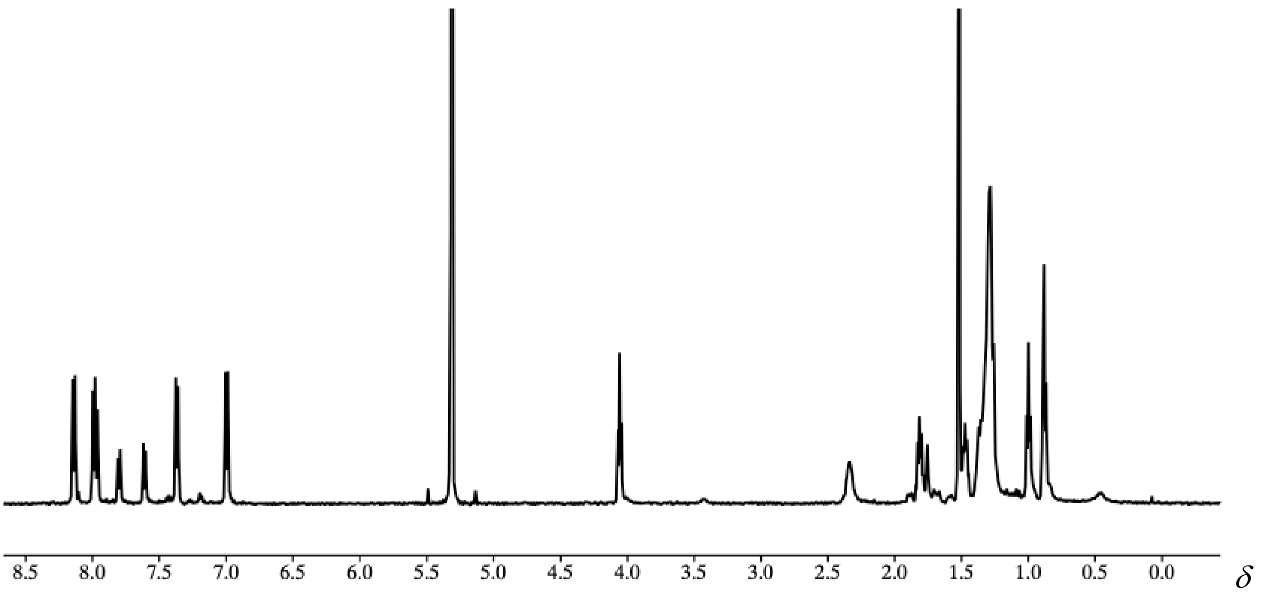

Figure S5. ^{1}H NMR spectrum of (+)-(*M*)-**2** in CD_2Cl_2 at 294 K. The small signals in the baseline are of the other isomer.

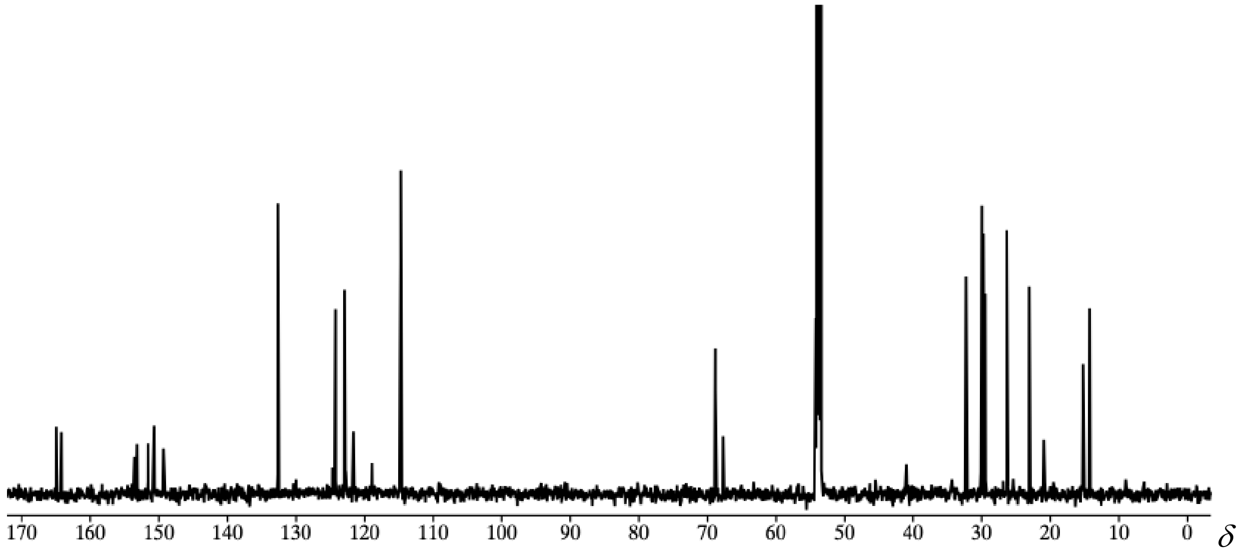

Figure S6. ^{13}C NMR spectrum of (+)-(*M*)-**2** in CD_2Cl_2 at 294 K.

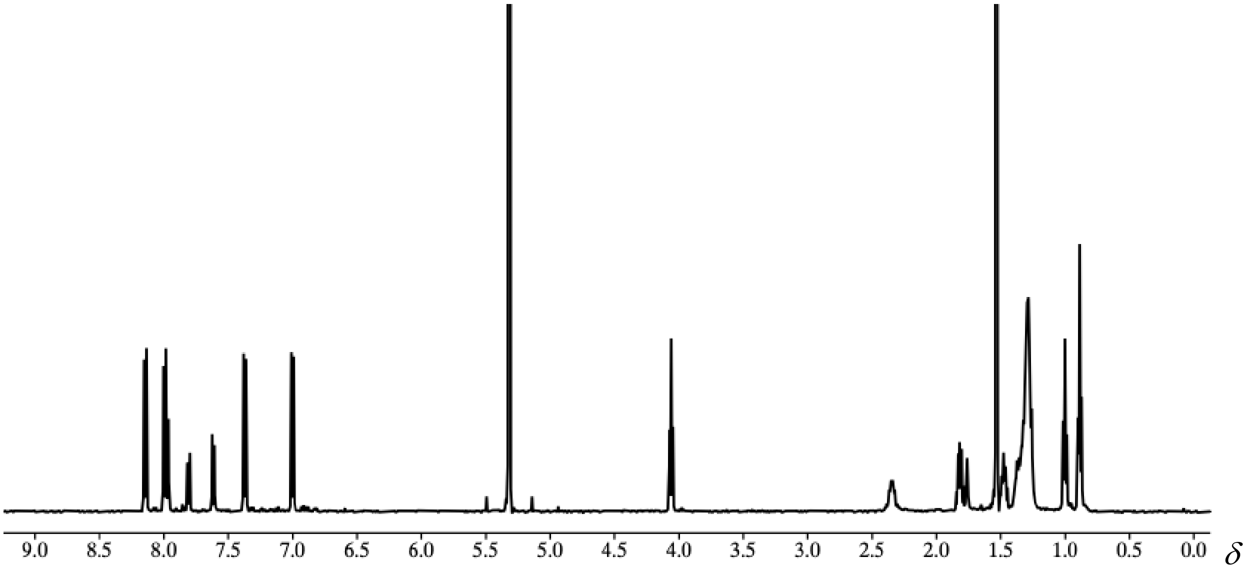

Figure S7. ^{1}H NMR spectrum of (-)-(*P*)-**2** in CD_2Cl_2 at 294 K.

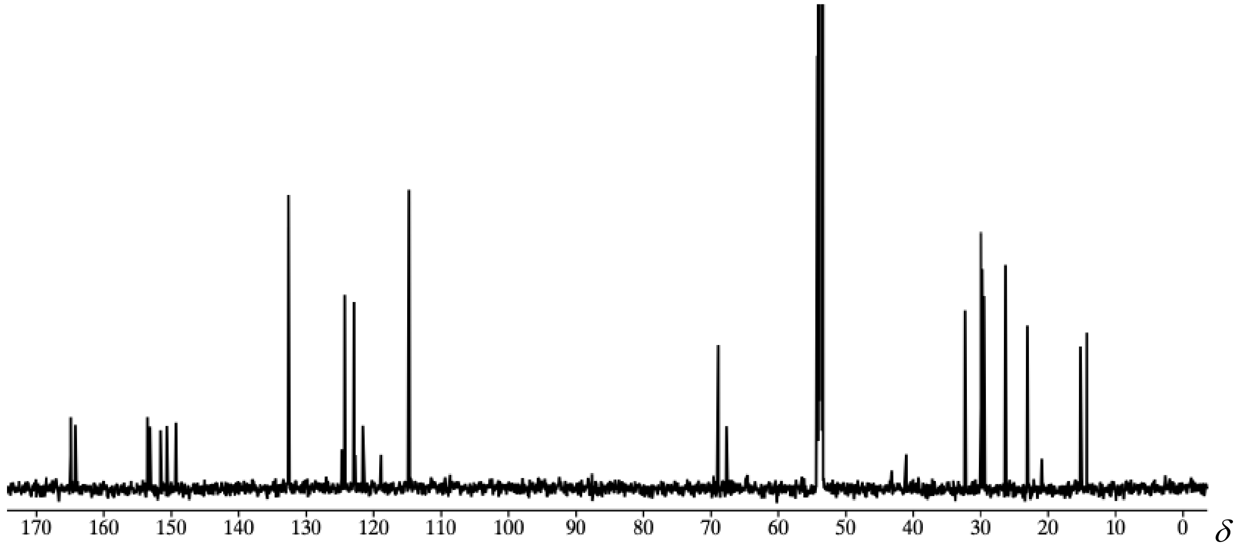

Figure S8. ^{13}C NMR spectrum of (-)-(*P*)-**2** in CD_2Cl_2 at 294 K.

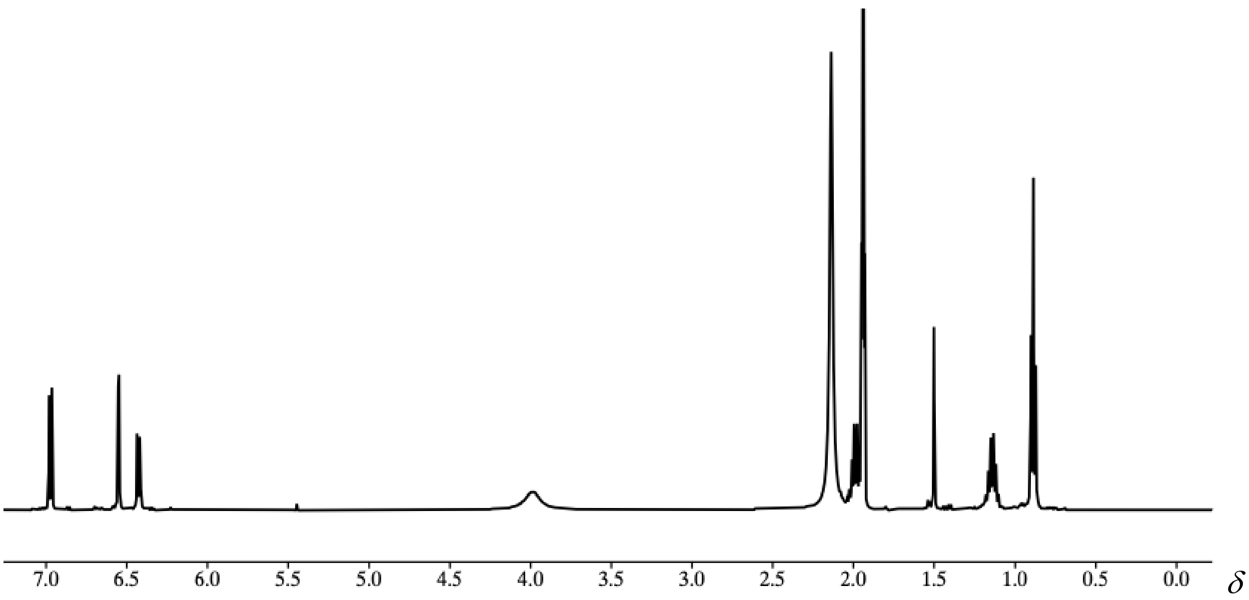

Figure S9. ^{1}H NMR spectrum of **3** in CD_3CN at 294 K.

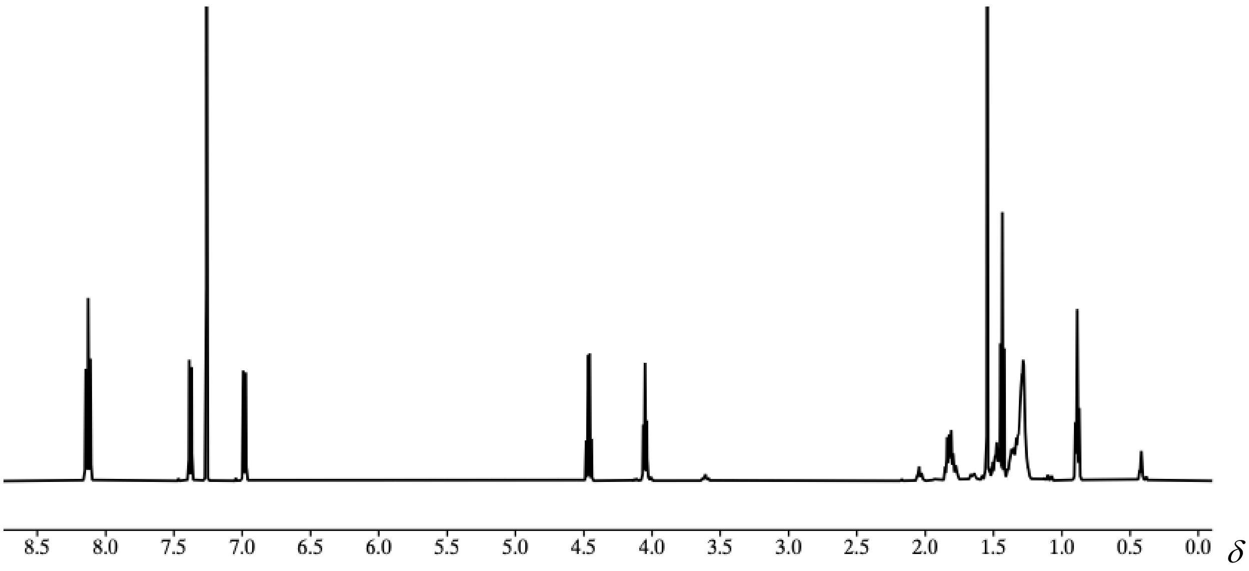

Figure S10. ^{1}H NMR spectrum of **4** in CD_3Cl at 294 K.

4. Photoisomerization Studies

UV-Vis and ^{1}H NMR spectroscopies were employed to study the photoisomerization of racemic compounds **1** and **2**. Spectrophotometric grade solvents were used for the absorption studies. Hydrazone and azobenzene-based switch solutions (3.0 mL, 1.0×10^{-5} M) in toluene were prepared and transferred into a 1.0 cm quartz cuvette for immediate UV/Vis absorption measurements. The solutions were then irradiated, and their UV spectra recorded. Isomerization cycles were measured by alternating the irradiation wavelength between the appropriate wavelengths and monitoring the change in UV/Vis absorption. The PSSs were determined upon continuous irradiation of the sample until no further isomerization was observed using ^{1}H NMR spectroscopy in toluene or methylene chloride.

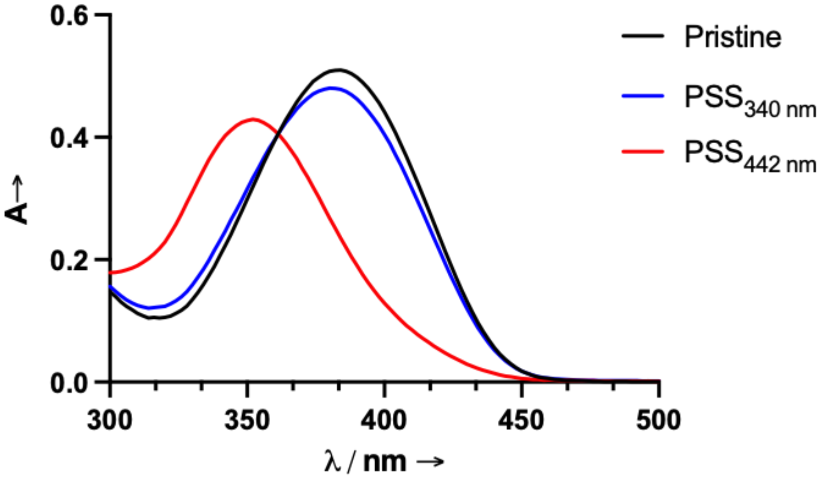

Figure S11. UV-Vis absorption spectra of **1**-*ZZZ* and **1**-*EEE* (1.0 x 10^{-5} M) in toluene: the UV spectrum of **1**-*EEE* was obtained upon 442 nm light irradiation of the pristine sample and **1**-*ZZZ* was obtained upon 340 nm light irradiation starting from PSS_{442}.

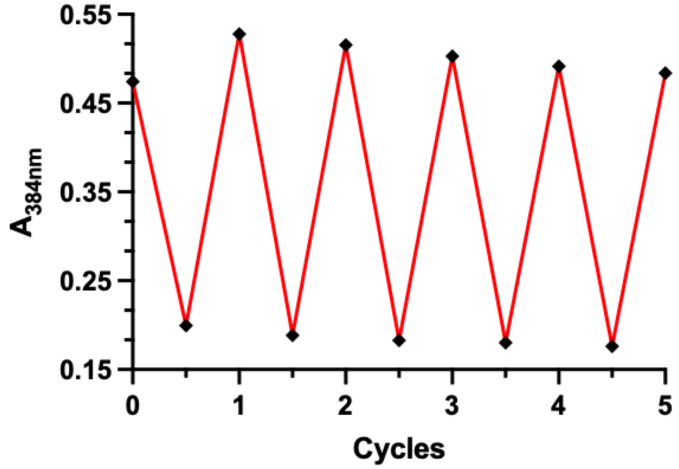

Figure S12. Photoisomerization cycles of **1** (1.0 x 10^{-5} M) in toluene at 294 K. The absorbance change at 384 nm was monitored while alternating the irradiation wavelength between 340 and 442 nm.

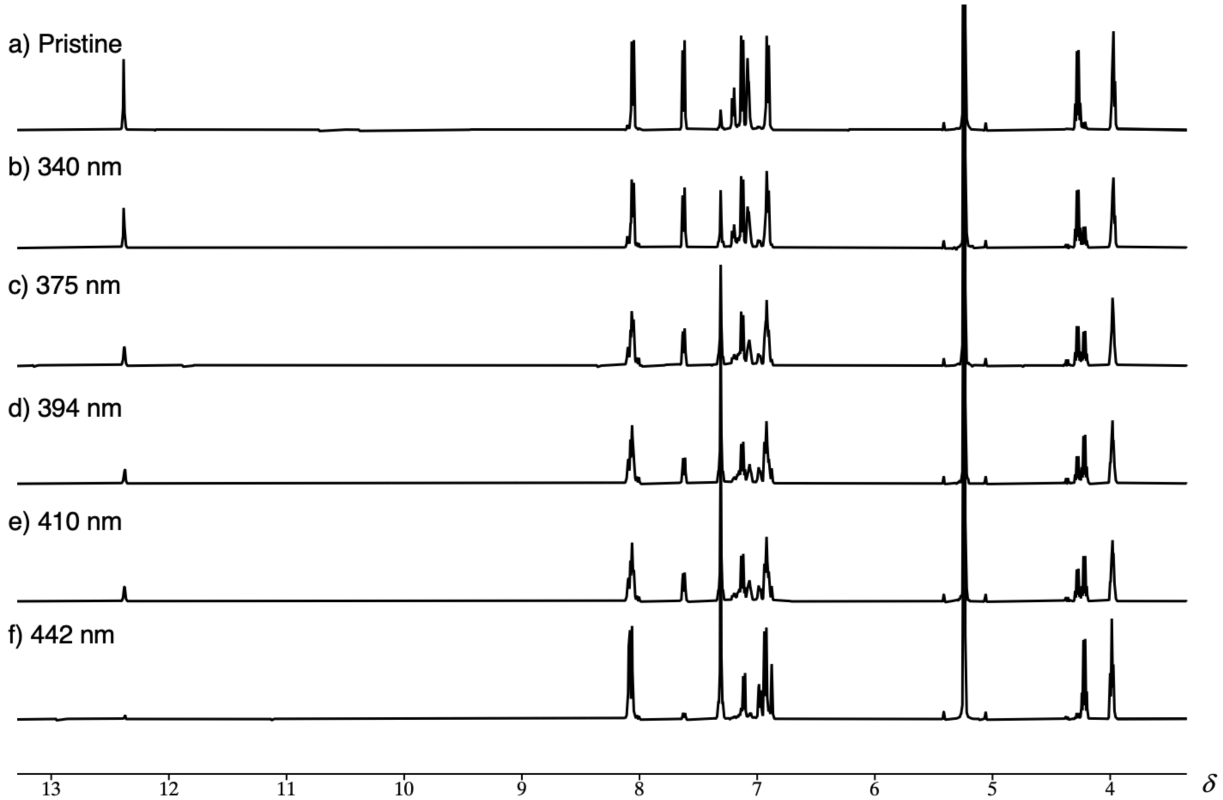

Figure S13: ^{1}H NMR spectra of (a) the pristine sample, (b) 340, (c) 375 nm, (d) 394 nm, (e) 410 nm, and (f) 442 nm photostationary states of **1** after sequential irradiation in CD_2Cl_2 at 294K.

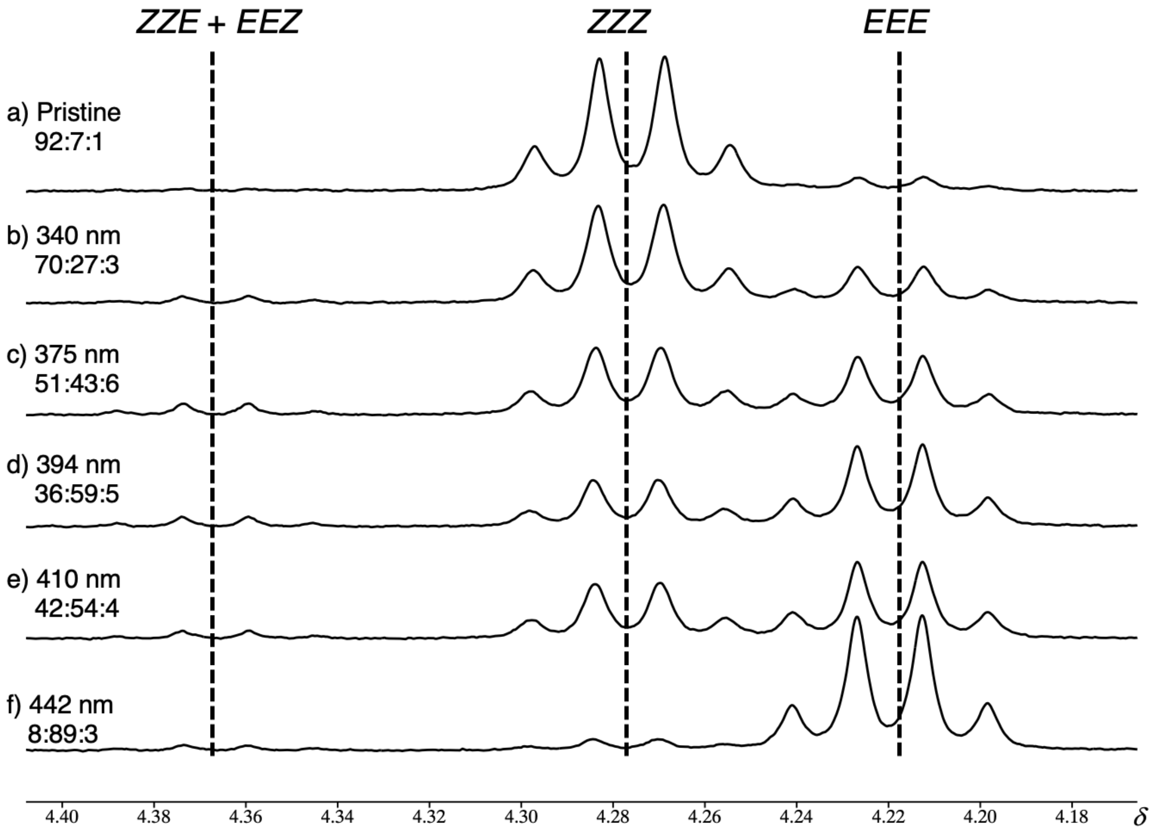

Figure S14: Zoom in of the ^{1}H NMR spectra of (a) the pristine sample, (b) 340, (c) 375 nm, (d) 394 nm, (e) 410 nm, and (f) 442 nm photostationary states of **1** after sequential irradiation in CD_2Cl_2 at 294K.

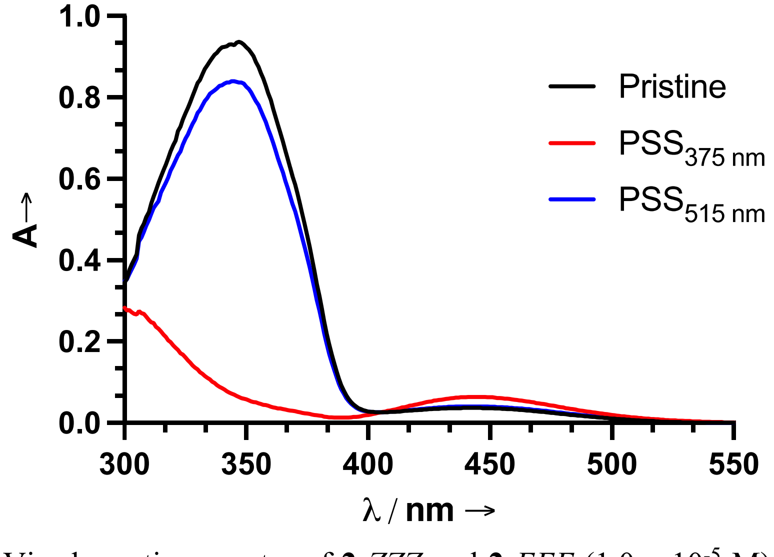

Figure S15. UV-Vis absorption spectra of **2**-*ZZZ* and **2**-*EEE* (1.0×10^{-5} M) in toluene: the UV spectrum of **1**-*ZZZ* was obtained upon 375 nm light irradiation of the pristine sample and **1**-*EEE* was obtained upon 515 nm light irradiation starting from PSS_{375}.

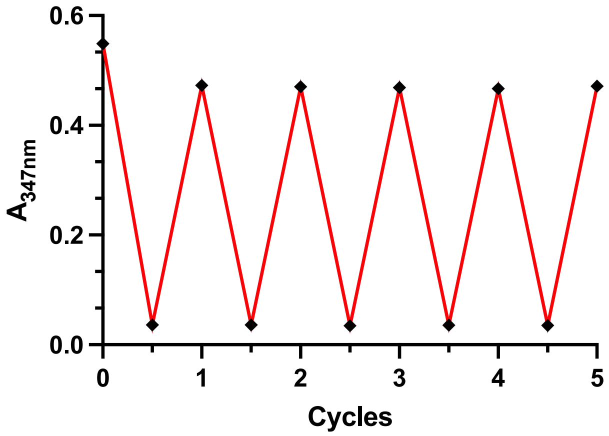

Figure S16. Photoisomerization cycles of **2** (1.0×10^{-5} M) in toluene at 294 K. The absorbance change at 347 nm was monitored while alternating the irradiation wavelength between 375 and 515 nm.

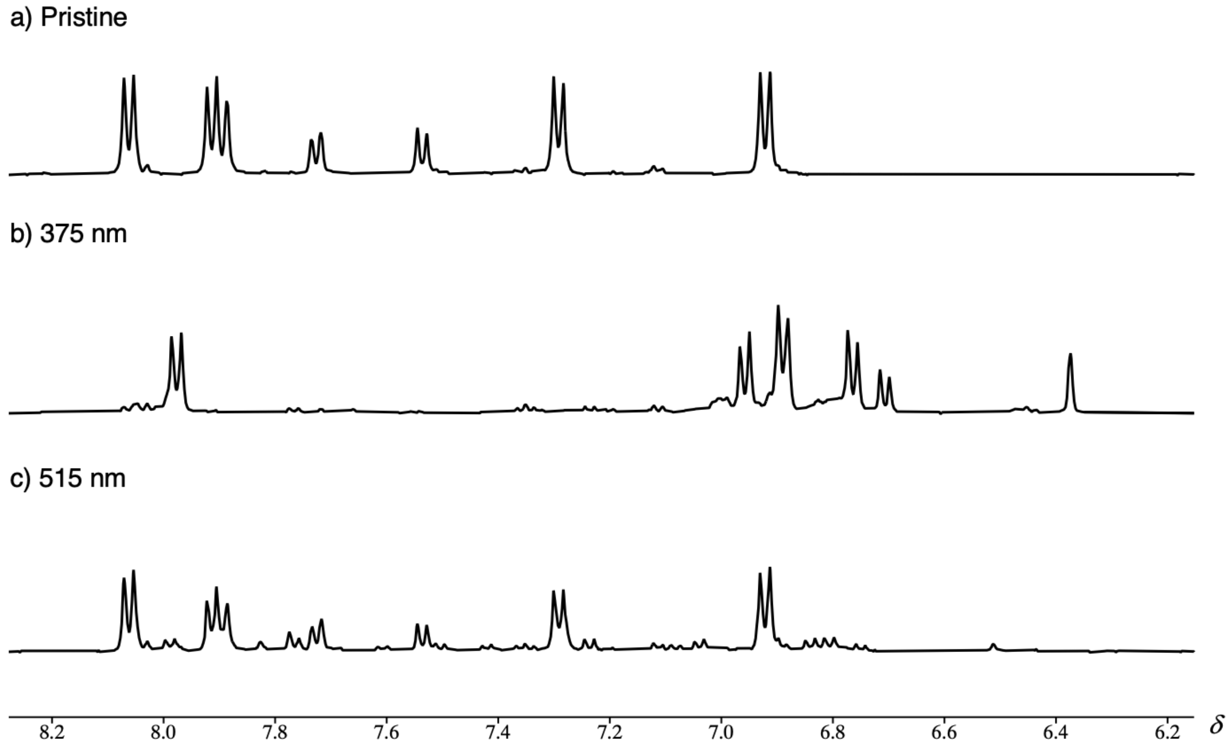

Figure S17: ^{1}H NMR spectra of (a) the pristine sample, (b) 375, and (c) 515 nm photostationary states of **2** after sequential irradiation in CD_2Cl_2 at 294K.

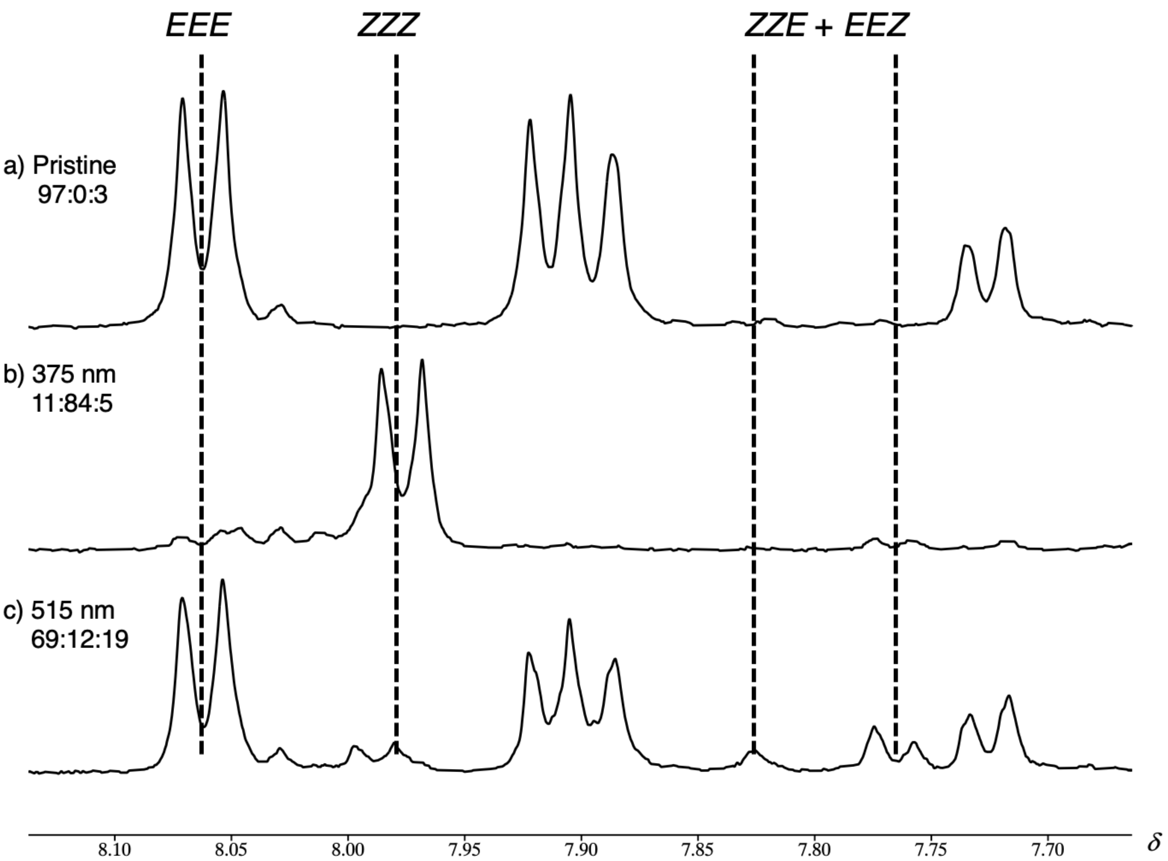

Figure S18: Zoom in of the ^{1}H NMR spectra of (a) the pristine sample, (b) 375, and (c) 515 nm photostationary states of **2** after sequential irradiation in CD_2Cl_2 at 294K.

5. Quantum Yield Determination

$$E \underset{k_{-1}}{\overset{k_1}{\rightleftharpoons}} Z$$

In a photochemical reaction, species ***E*** absorbs light to generate product ***Z***. The general kinetics of a basic photochemical reaction can be expressed using Eq. 1[S3]

$$\frac{dC_t}{dt} = k_1(C_o - C_t) - k_{-1}C_t \quad \text{Eq. 1}$$

The rates of the forward and reverse processes (k_1 and k_{-1}), which is related to the quantum yield, the observed first-order rate constant, molar photon flux, and the measurable properties of sample ***E*** and ***Z***, can be expressed using Eqs. 2 and 3

$$k_1 = \frac{I_0 l(1-10^{-A(t)})\Phi_{E\to Z}\varepsilon_E}{VA(t)}; \; k_{-1} = \frac{I_0 l(1-10^{-A(t)})\Phi_{Z\to E}\varepsilon_Z}{VA(t)} \qquad \text{Eqs. 2 and 3}$$

where $\Phi_{E\to Z}$ is the photoisomerization yield going from ***E*** to ***Z***; k_1 represents the rate constant (obtained from the exponential fit of a graph of *Abs* vs. time); V indicates sample volume; I_0 indicates molar photon flux; ε_E indicates molar absorption coefficient of sample ***E***, l indicates light path length, and $\frac{1-10^{-A(t)}}{A(t)}$ indicates the photokinetic factor.

At the photostationary state, the change in reaction rate as a function of time approaches a value of zero, and after substitution of k_1 and k_{-1}, the quotient of the quantum yield values of the forward and reverse processes can be expressed using Eq. 4

$$\frac{\Phi_{E\to Z}}{\Phi_{Z\to E}} = \frac{C_\infty \varepsilon_Z}{(C_O - C_\infty)\varepsilon_E} \qquad \text{Eq. 4}$$

Defining the formation of the *Z* isomer as a $y = C_t / C_0$, where C_t represents the fraction of Z isomer and C_0 represents the initial fraction of *E* isomer, Eqs. 1–4 were combined and can be expressed using Eq. 5

$$ln\frac{y_\infty - y}{y_\infty - y_0} = -\frac{I_0 l \Phi_{E\to Z}\varepsilon_E}{V y_\infty}\int_0^t \frac{1-10^{-A(t)}}{A(t)}dt \quad \text{Eq. 5}$$

where $y_\infty = C_\infty / C_0$ is the fraction of *Z* isomer at PSS, $B = \frac{I_0 l \Phi_{E\to Z}\varepsilon_E}{V y_\infty}$, and $X(t) = \int_0^t \frac{1-10^{-A(t)}}{A(t)}dt$ represents the integrated photokinetic factor.[S4] Plotting the fraction of the *Z* isomer against $x(t)$ can be expressed using Eq. 6 and following exponential fitting the *B* and y_∞ values can be determined.

$$y = (y_0 - y_\infty)e^{-Bx(t)} + y_\infty \quad \text{Eq. 6}$$

The quantum yield, $\Phi_{E\to Z}$ can then be calculated by rearranging the term for *B* and be expressed by Eq. 7

$$\Phi_{E\to Z} = \frac{BVy_{\infty}}{I_0 l \varepsilon_E} \quad \text{Eq. } 7^{S5}$$

The molar photon flux I_0 at 340, 375, and 442 nm were determined using chemical actinometry (1.62 x 10^{-7}, 1.09 x 10^{-7}, and 1.22 x 10^{-7} $mol{\cdot}s^{-1}$, respectively).[S6] A 0.002 L (= V_0) solution of potassium ferrioxalate (0.06 M) in 0.05 M H_2SO_4 was placed in an 1.0 cm cuvette and irradiated for 30 s (= t_0). The irradiated solution was combined with 3.5 equiv. of ferrozine and stirred under dark for an hour. The resulting solution, containing reddish-purple $[Fe(ferrozine)_3]^{2+}$ complex was diluted by a factor of 30 (= n), and its absorbance was measured at 563 nm (A_{563}), where its molar absorption coefficient (ε_{563}) is 27,900 cm^{-1} M^{-1}. The molar photon flux I_0 of the light source at different wavelengths was determined using Eq. 8.

$$I_0\left(mol \cdot s^{-1}\right) = \frac{A_{563} \cdot n \cdot V_0}{\varepsilon_{563} \cdot l \cdot t_0 \cdot \phi_{\lambda}} \quad \text{Eq. 8}$$

where l indicates the length of the cuvette, and ϕ_{λ} stands for the quantum yield of the photo-reduction of Fe(III) oxalate induced by the light source (ϕ_{340} = 1.25; ϕ_{375} = 1.21; ϕ_{442} = 1.11). The molar photon flux for 515 nm was determined with a Thorlabs optical power meter, (3.01 x 10^{-7} $mol{\cdot}s^{-1}$). The Φ for the racemic compounds **1** and **2** were measured, and their photophysical properties are listed in Tables S1 and S2.

Table S1. Photophysical properties of compound **1.**

	λ_{irr} (nm) / ε ($M^{-1}cm^{-1}$)		Φ		
	Z	*E*	*E* → *Z*	*Z* → *E*	PSS_{340} / PSS_{442}
**1**	384 / 87,700	347 / 76,400	1.8 ± 0.1%	2.2 ± 0.1%	81:19 / 9:91

Table S2. Photophysical properties of compound **2.**

	λ_{irr} (nm) / ε ($M^{-1}cm^{-1}$)		Φ		
	*π–π**	*n–π**	*E* → *Z*	*Z* → *E*	PSS_{375} / PSS_{515}
**2**[a]	347 / 93,600	443 / 6,400	11.6 ± 1.3%	23.0 ± 0.9%	87:13 / 21:79

[a] The *π–π** and the *n–π** peaks were determined from the *EEE* and *ZZZ* isomers respectively.

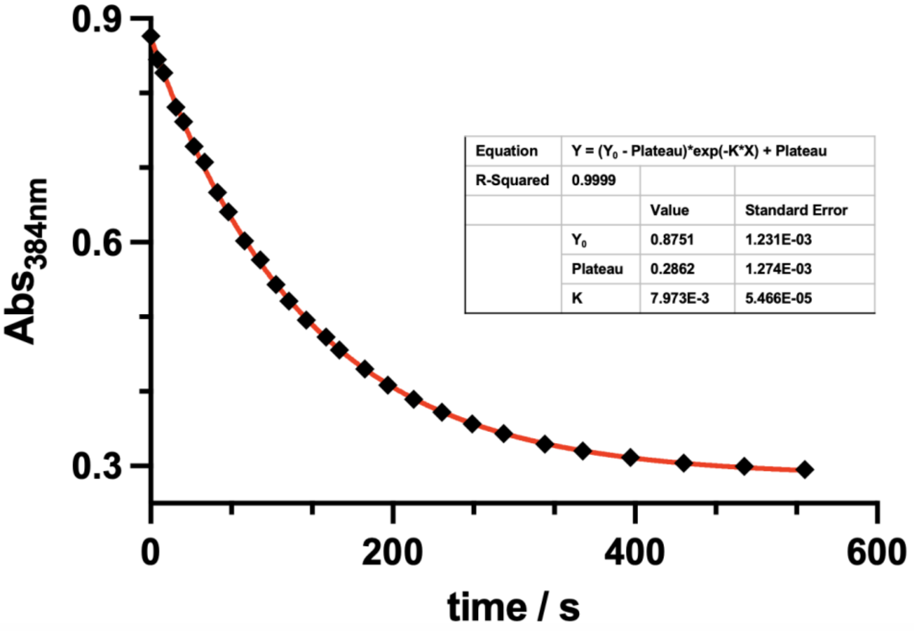

Figure S19: Kinetics of the photoisomerization (irradiation at 442 nm) of **1**-*ZZZ* to **1**-*EEE* in toluene (1.0×10^{-5} M) at 298 K; the plot is of the absorbance (λ_{max} = 384 nm) of **1**-*ZZZ* as a function of time. $\varepsilon_{1\text{-}ZZZ@442nm}$ = 7,530 $M^{-1}\cdot cm^{-1}$ was used for the quantum yield calculations. The photoisomerization quantum yield was calculated to be 2.2 ± 0.1% based on three consecutive measurements.

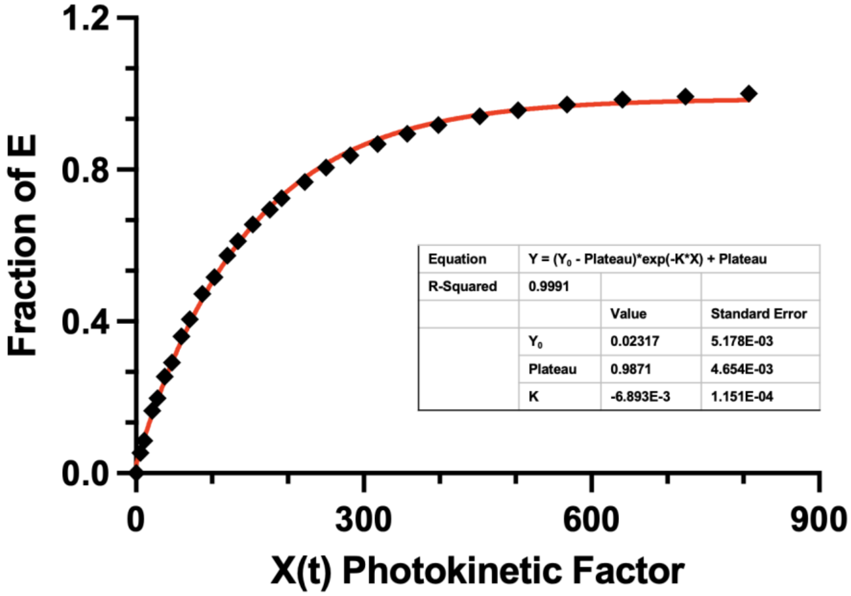

Figure S20: Kinetics for the photoisomerization (irradiation at 442 nm) of **1**-*ZZZ* to **1**-*EEE* in toluene (1.0×10^{-5} M) at 298 K; the plot is of the fraction of **1**-*ZZZ* as a function of the photokinetic factor, X(t).

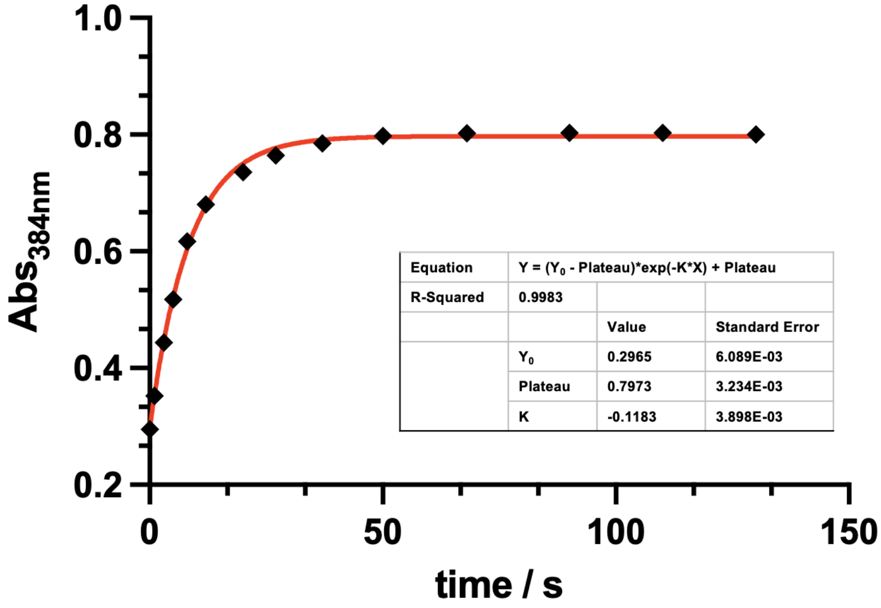

Figure S21: Kinetics of the photoisomerization (irradiation at 340 nm) of **1**-*EEE* to **1**-*ZZZ* in toluene (1.0×10^{-5} M) at 298 K; the plot is of the absorbance (λ_{max} = 384 nm) of **1**-*EEE* as a function of time. $\varepsilon_{1\text{-}EEE@340nm}$ = 72,300 $M^{-1} \cdot cm^{-1}$ was used for the quantum yield calculations. The photoisomerization quantum yield was calculated to be 1.8 ± 0.1% based on three consecutive measurements.

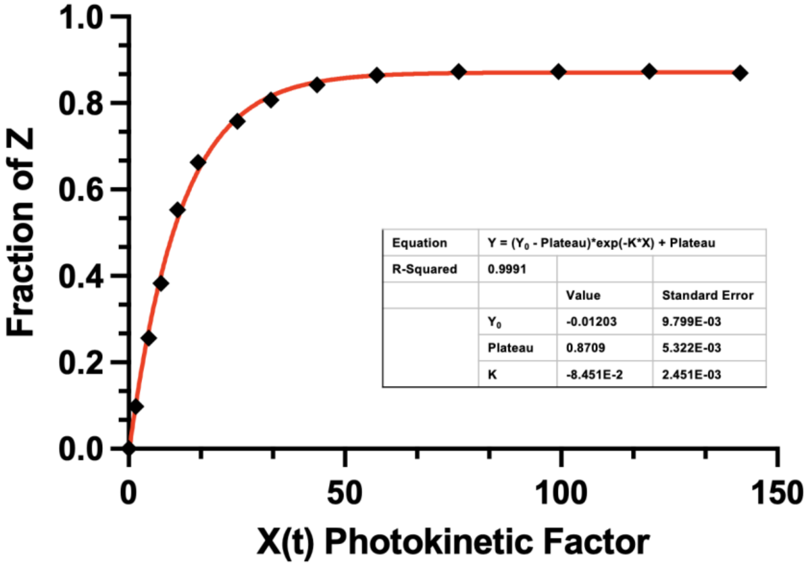

Figure S22: Kinetics of the photoisomerization (irradiation at 340 nm) of **1**-*EEE* to **1**-*ZZZ* in toluene (1.0×10^{-5} M) at 298 K; the plot is of the fraction of **1**-*ZZZ* as a function of the photokinetic factor, X(t).

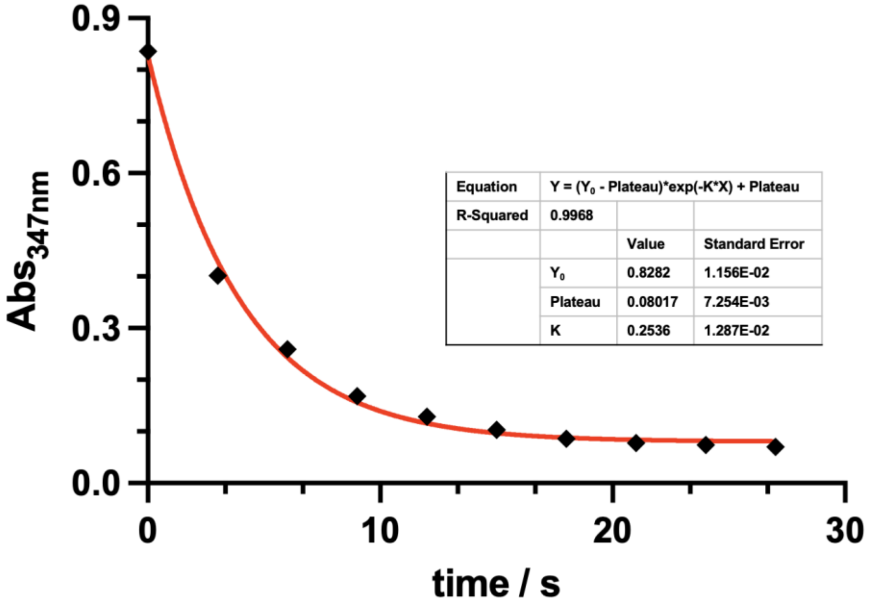

Figure S23: Kinetics of the photoisomerization (irradiation at 375 nm) of **2**-*EEE* to **2**-*ZZZ* in toluene (1.0×10^{-5} M) at 298 K; the plot is of the absorbance (λ_{max} = 347 nm) of **2**-*EEE* as a function of time. $\varepsilon_{2\text{-}EEE@375nm}$ = 46,000 $M^{-1}\cdot cm^{-1}$ was used for the quantum yield calculations. The photoisomerization quantum yield was calculated to be 11.6 ± 1.3% based on three consecutive measurements.

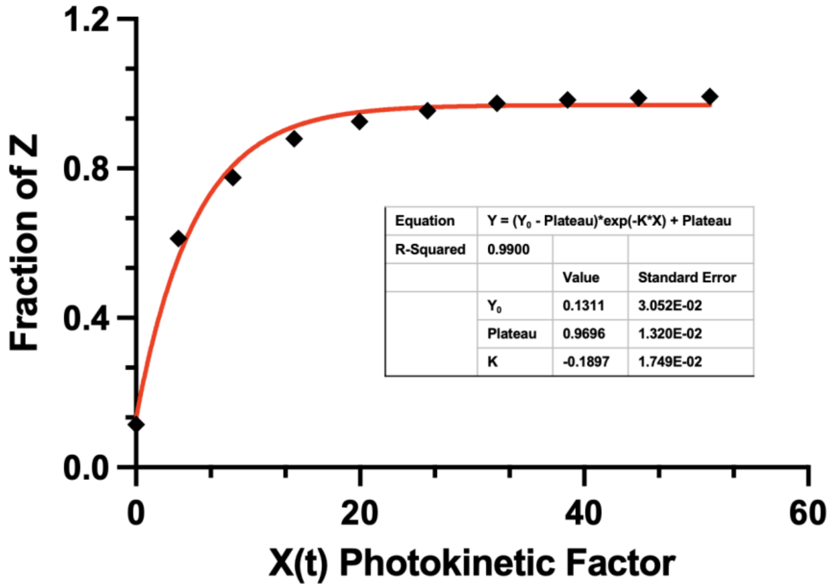

Figure S24: Kinetics for the photoisomerization (irradiation at 375 nm) of **2**-*EEE* to **2**-*ZZZ* in toluene (1.0×10^{-5} M) at 298 K; the plot is of the fraction of **2**-*ZZZ* as a function of the photokinetic factor, X(t).

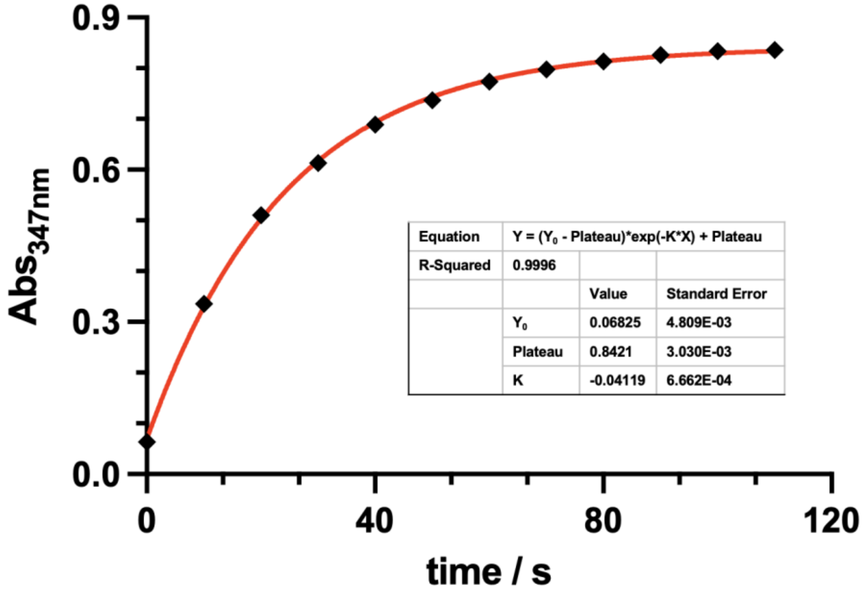

Figure S25: Kinetics of the photoisomerization (irradiation at 515 nm) of **2**-*ZZZ* to **2**-*EEE* in toluene (1.0×10^{-5} M) at 298 K; the plot is of the absorbance (λ_{max} = 347 nm) of **2**-*EEE* as a function of time. $\varepsilon_{2\text{-}ZZZ@515nm}$ = 1,040 $M^{-1}\cdot cm^{-1}$ was used for the quantum yield calculations. The photoisomerization quantum yield was calculated to be 23.0 ± 0.9% based on three consecutive measurements.

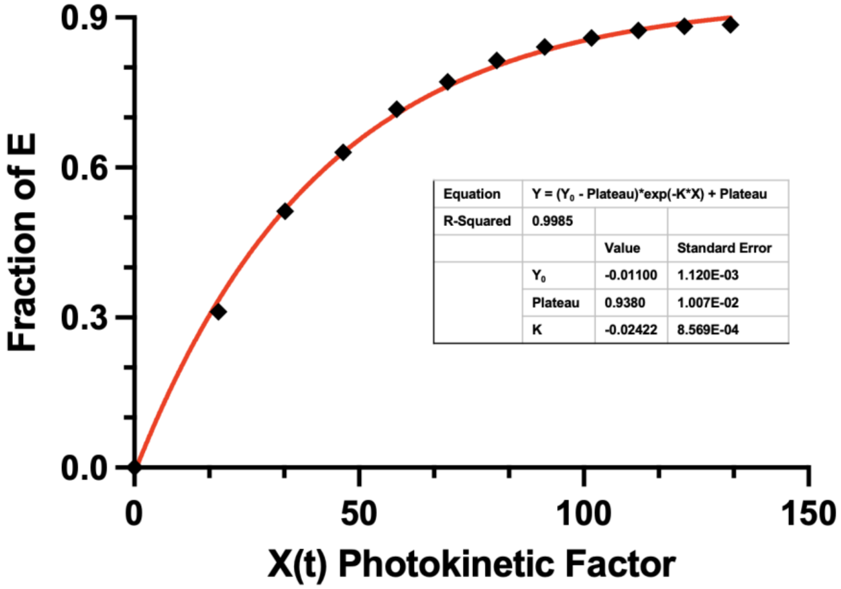

Figure S26: Kinetics for the photoisomerization (irradiation at 515 nm) of **2**-*ZZZ* to **2**-*EEE* in toluene (1.0×10^{-5} M) at 298 K; the plot is of the fraction of **2**-*EEE* as a function of the photokinetic factor, X(t).

6. Circular Dichroism Measurements

The CD spectra of the chiral dopants **1** and **2** and racemic mixture of **1** and 2 were recorded at 20 °C on a J815 CD Spectrometer in THF at a concentration of 2.5 × 10^{-4} M. The CD spectra of the starting chiral amine, **3**, and its racemic mixture are also given for comparison.

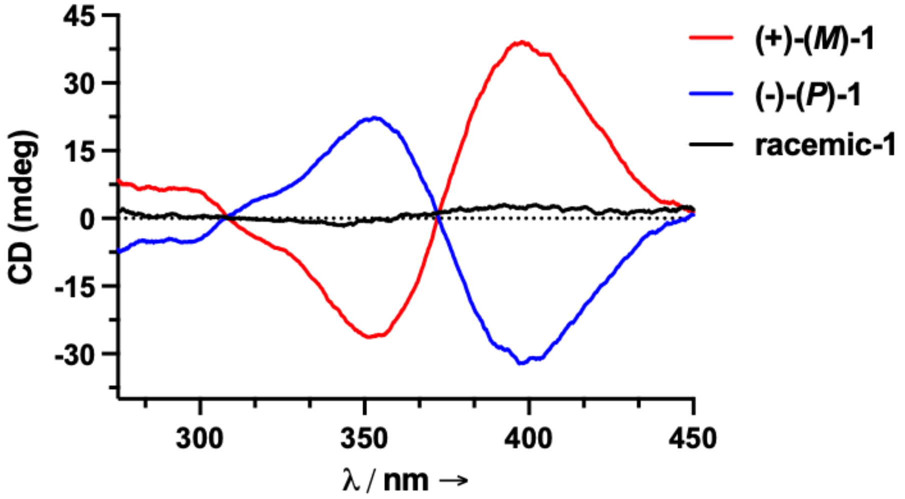

Figure S27: CD spectra in THF (2.5 × 10^{-4} M) of the chiral molecular switches (-)-(*P*)-**1** and (+)-(*M*)-**1** compared to the racemic mixture of **1.**

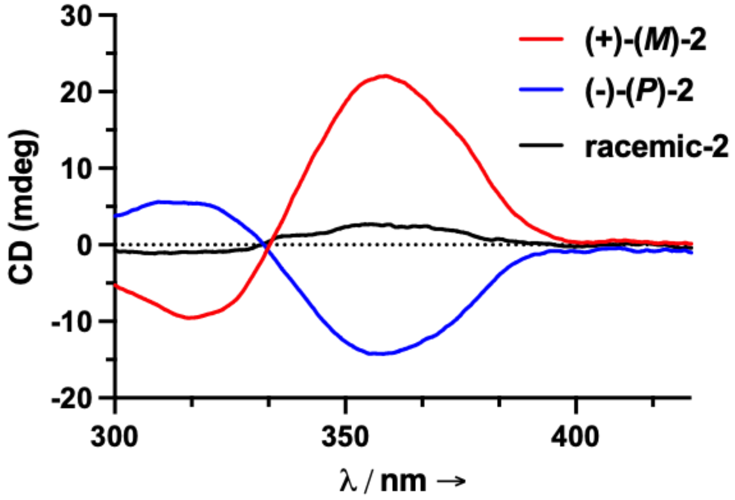

Figure S28: CD spectra in THF (2.5 × 10^{-4} M) of the chiral molecular switches (-)-(*P*)-**2** and (+)-(*M*)-**2** compared to the racemic mixture of **2**.

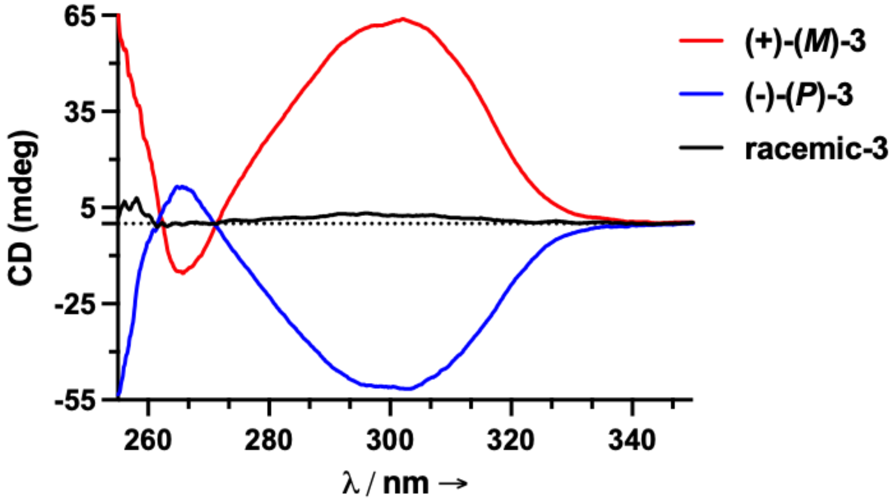

Figure S29: CD spectra in THF (2.5×10^{-4} M) of (-)-(*P*)-**3** and (+)-(*M*)-**3** compared to the racemic mixture of **3**.

7. Thermal Isomerization Half-Life Determination

The thermal isomerization kinetics of the azobenzene and hydrazone-based dopants were studied at 292 and 373 K respectively using ^{1}H NMR spectroscopy. Solutions (~10^{-4} M) of the switches in 0.5 mL toluene-d_8 were irradiated at a specific wavelength and then left in a Haake F3 circulating water bath at a preset temperature. ^{1}H NMR spectra were then acquired at different intervals at room temperature to monitor the change in proton signal intensity as a function of time. The thermal isomerization rates (k_1) were determined by least-square curve fittings using an integrated and combined rate equation (Eq. 9) of a single-species reversible reaction:

$$\mathbf{A} \underset{k_{-1}}{\overset{k_1}{\rightleftharpoons}} \mathbf{Z}$$

$$K_{eq} = \frac{k_1}{k_{-1}} = \frac{C_A^0 - C_A^{eq}}{C_A^{eq}} \qquad \text{Eq. 9}$$

$$\ln\left(\frac{C_A - C_A^{eq}}{C_A^0 - C_A^{eq}}\right) = -(k_1 + k_{-1})t \qquad \text{Eq. 10}$$

Combining Eq. 9 and 10 will give Eq. 11 as follow,

$$C_A = \left(C_A^0 - C_A^{eq}\right) \times e^{\left(\frac{-k_1 C_A^0 t}{C_A^0 - C_A^{eq}}\right)} + C_A^{eq} \qquad \text{Eq. 11}$$

where C_A, C_A^0, and C_A^{eq} stand for experimental, initial, and equilibrium concentrations of the metastable configuration of the hydrazone switch, respectively; *t* stands for the thermal relaxation time.

For hydrazones dopants, the resulting k_1 values elevated temperatures were measured and then used to calculate the energy barriers for the *E*→*Z* thermal relaxation using the Eyring equation (Eq. 12) With the energy barriers ($\Delta G^{\ddagger}$) in hand, the Arrhenius equation (Eq. 13) was used to extrapolate the rate constants (k_2) at 298K, from which the room-temperature thermal half-lives were determined.[S7]

$$\Delta G^{\ddagger} = 8.314 \times T \times \left[23.760 + \ln\left(\frac{T}{k}\right)\right] \qquad \text{Eq. 12}$$

Where $\Delta G^{\ddagger}$, *T* and *k* refer to energy barrier for thermal relaxation (J mol^{-1}), temperature (K) and rate constant (s^{-1}).

$$\ln\left(\frac{k_2}{k_1}\right) = \frac{\Delta G^{\ddagger}}{R} \times \left(\frac{1}{T_1} - \frac{1}{T_2}\right) \qquad \text{Eq. 13}$$

Where k_1 and k_2 refer to the rate constants at elevated temperature (292 and 373 K) and room temperature (298 K), respectively; T_1 and T_2 refer to the elevated temperature and room temperature; R refers to ideal gas constant (J K^{-1} mol^{-1}).

Table S3. Thermal kinetics data for **1** and **2** at 298 K.

Dopant	$\Delta G^{\ddagger}$ (kcal/mol)	k (s^{-1})	$\tau_{1/2}$
**1**	28.9 ± 0.1	4.9 x 10^{-9} ± 6.4 x 10^{-10}	4.5 ± 0.6 years
**2**	23.0 ± 0.1	1.5 x 10^{-6} ± 9.0 x 10^{-8}	5.6 ± 0.4 days

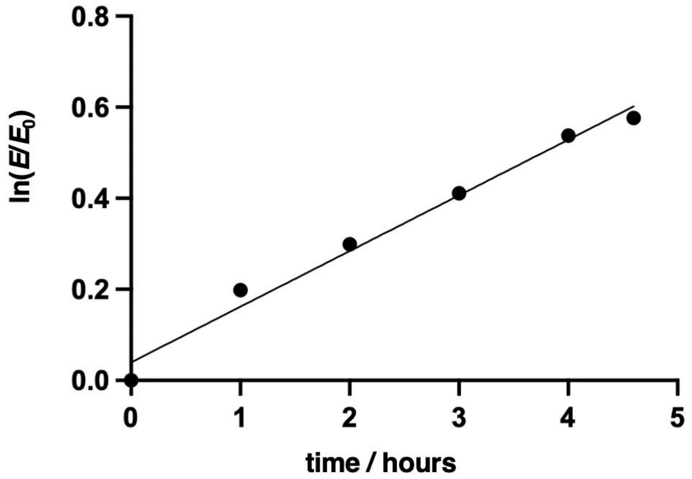

Figure S30: Thermal isomerization of **1**-*EEE* → **1**-*ZZZ* in toluene-d_8 at 298 K. The plot is of the concentration of **1**-*EEE* as a function of time. The resulting k_1 value was calculated to be (4.9 ± 0.64) × 10^{-9} s^{-1} based on three consecutive measurements at room temperature.

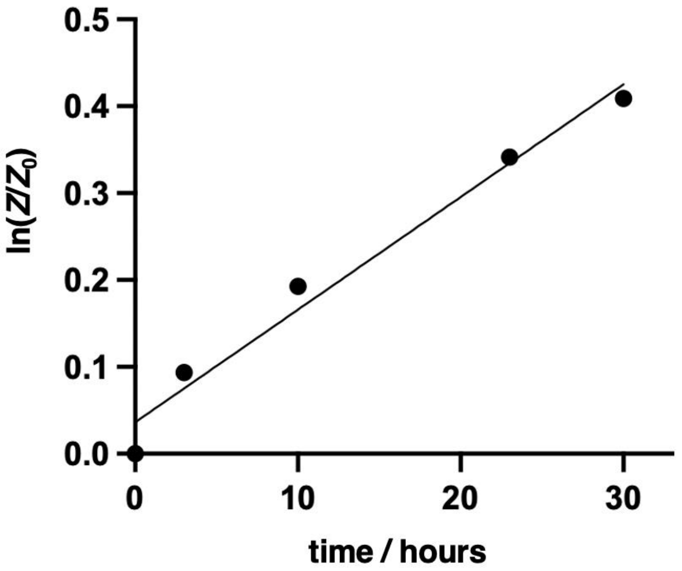

Figure S31: Thermal isomerization of **2**-*ZZZ* → **2**-*EEE* in toluene-d_8 at 298 K. The plot is of the concentration of **2**-*ZZZ* as a function of time. The resulting k_1 value was calculated to be $(1.5 \pm 0.09) \times 10^{-6}$ s^{-1} based on three consecutive measurements at room temperature.

8. HTP and Reflectance of Adaptive Films

Liquid crystalline samples were prepared by mixing the appropriate amount of liquid crystalline host, 5CB, with photochromic dopants **1** and **2**. Even mixing was achieved by dissolving the mixture in spectroscopic grade methylene chloride and evaporating it under reduced pressure, followed by heating to the isotropic temperature and slow cooling to room temperature. Low concentration mixtures (1.0 mol% or less) were loaded into wedge cells (EHC KCRK-03, -05, -07, -11) at room temperature to check which gave the best alignment. The helical twisting power (β) was calculated by the Grandjean-Cano wedge method.[S8,9] Briefly, the pitch (p) is proportional to the distance between disclination lines (s) and the angle of the wedge (θ) (equation 12). The β is given by the inverse of pitch times concentration (equation 13. To demonstrate the ability to kinetically trap the helical assembly at a particular pitch based on irradiation wavelength, photochromic samples in 5CB were irradiated with 340/442 and 375/515 nm light until the PSS was reached and the β measured. The PSS of the mixture was determined as the point at which the helical pitch no longer changed with continued irradiation.

$$p = 2 \cdot s \cdot \tan(\theta) \qquad \text{Eq. 12}$$

$$\beta = \frac{1}{c \cdot p} \qquad \text{Eq. 13}$$

High concentration mixtures (> 1.0 mol%) were loaded into 5 μm planar cells (Instec LC3-5.0) to measure the reflectance of adaptive films.

Table S4. β values of **1** and **2** in 5CB.

	$\beta_{pristine}$ (μm^{-1})	β_{ZZZ} (μm^{-1})	β_{EEE} (μm^{-1})	$\Delta\beta$ (μm^{-1})
(+)-(*M*)-**1**	+147	+145	+119	+28
(-)-(*P*)-**1**	–145	–142	–116	–29
(+)-(*M*)-**2**	+93	< +10	+51	+83
(-)-(*P*)-**2**	–93	< –10	–55	–83

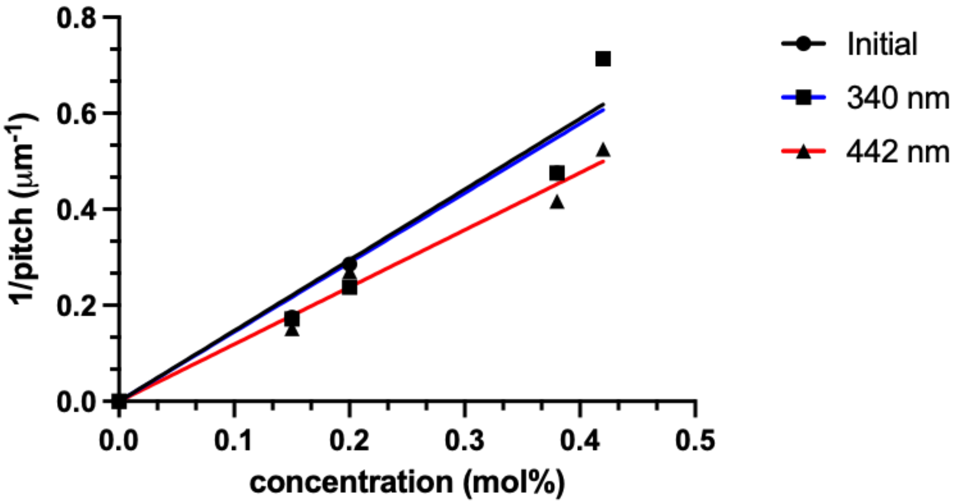

Figure S32: Plots of 1/p versus concentration of (+)-(*M*)-**1** in 5CB of the pristine state and at PSS_{340} and PSS_{442}.

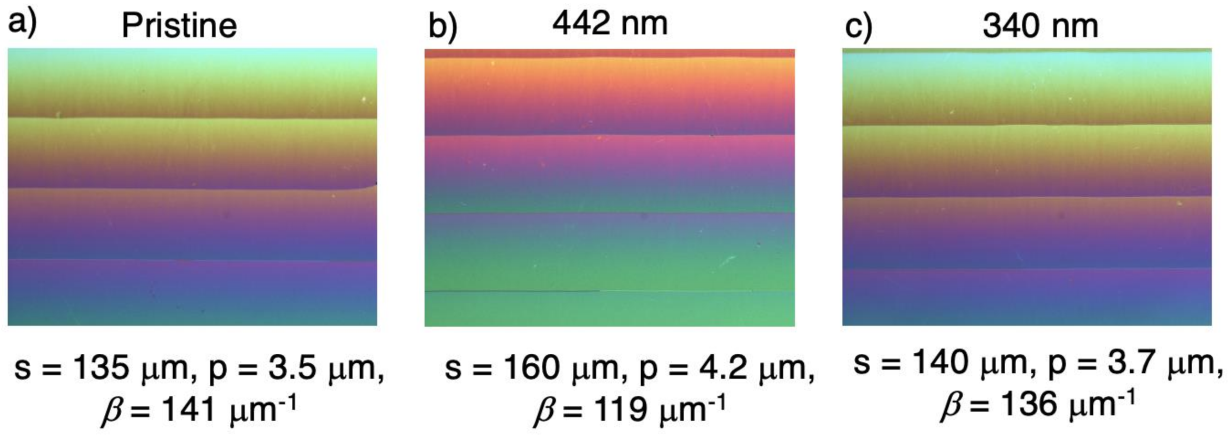

Figure S33: Photomicrographs of (+)-(*M*)-**1** (0.20 mol%) in 5CB after irradiating (a) the pristine state with (b) 442 and (c) 340 nm light in a KCRK05 cell.

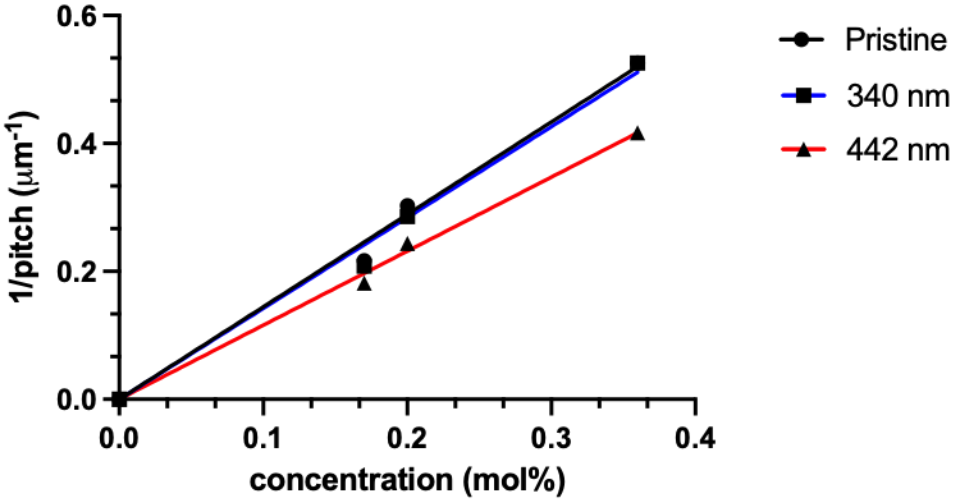

Figure S34: Plots of 1/p versus concentration of (-)-(*P*)-**1** in 5CB of the pristine state, and at PSS_{340} and PSS_{442}.

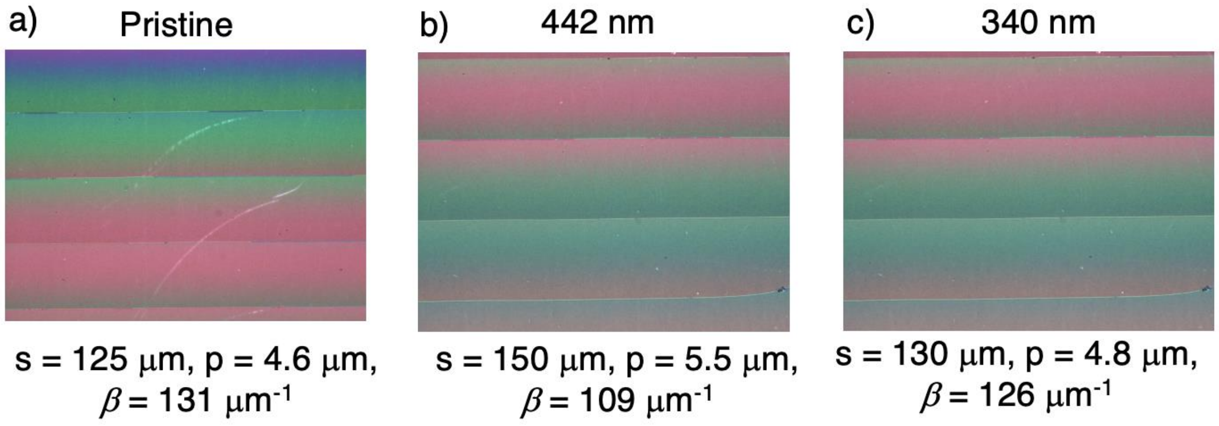

Figure S35: Photomicrographs of (-)-(*P*)-**1** (0.17 mol%) in 5CB after irradiating (a) the pristine state with (b) 442 and (c) 340 nm light in a KCRK07 cell.

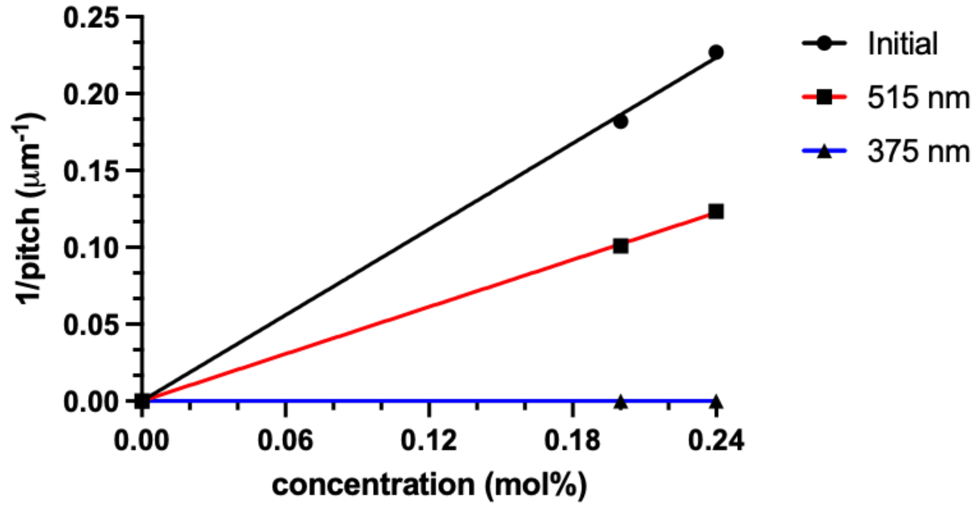

Figure S36: Plots of 1/p versus concentration of (+)-(*M*)-**2** in 5CB of the pristine state, and at PSS_{375} and PSS_{515}.

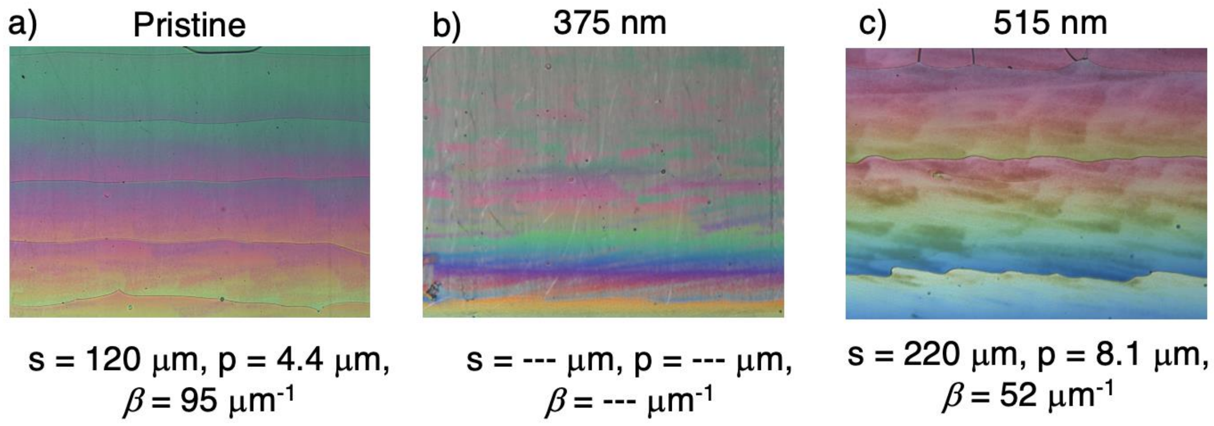

Figure S37: Photomicrographs of (+)-(*M*)-**2** (0.20 mol%) in 5CB after irradiating (a) the pristine state with (b) 375 and (c) 515 nm light in a KCRK07 cell.

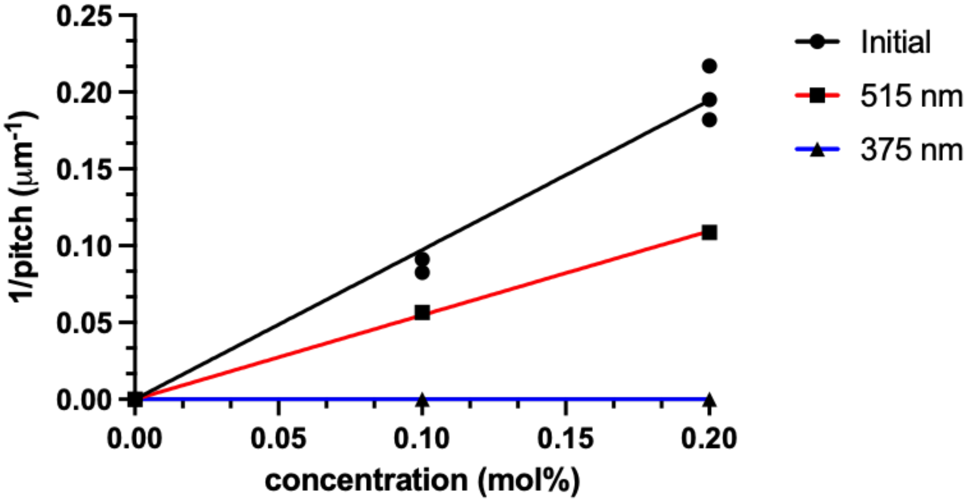

Figure S38: Plots of 1/p versus concentration of (-)-(*P*)-**2** in 5CB of the pristine state, and at PSS_{375} and PSS_{515}.

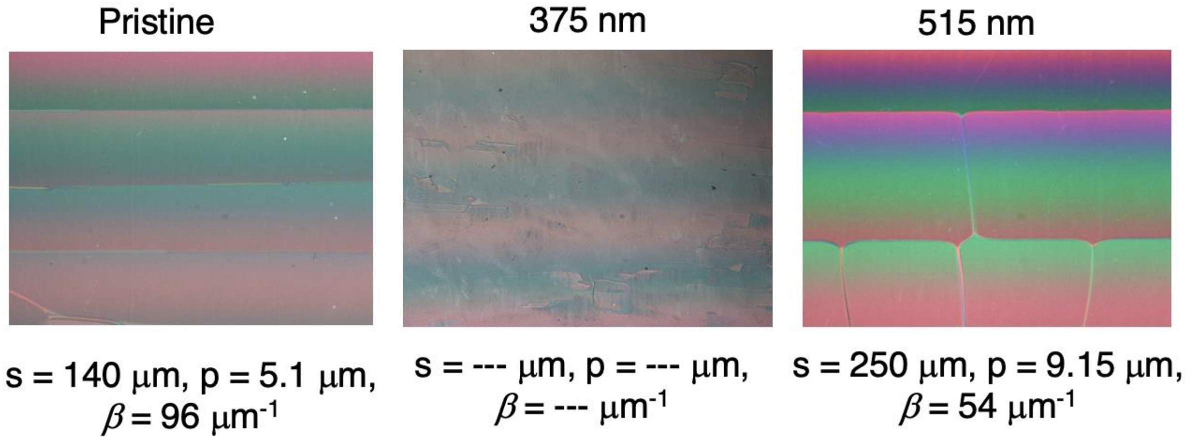

Figure S39: Photomicrographs of (-)-(*P*)-**2** (0.20 mol%) in 5CB after irradiating (a) the pristine state with (b) 375 and (c) 515 nm light in a KCRK07 cell.

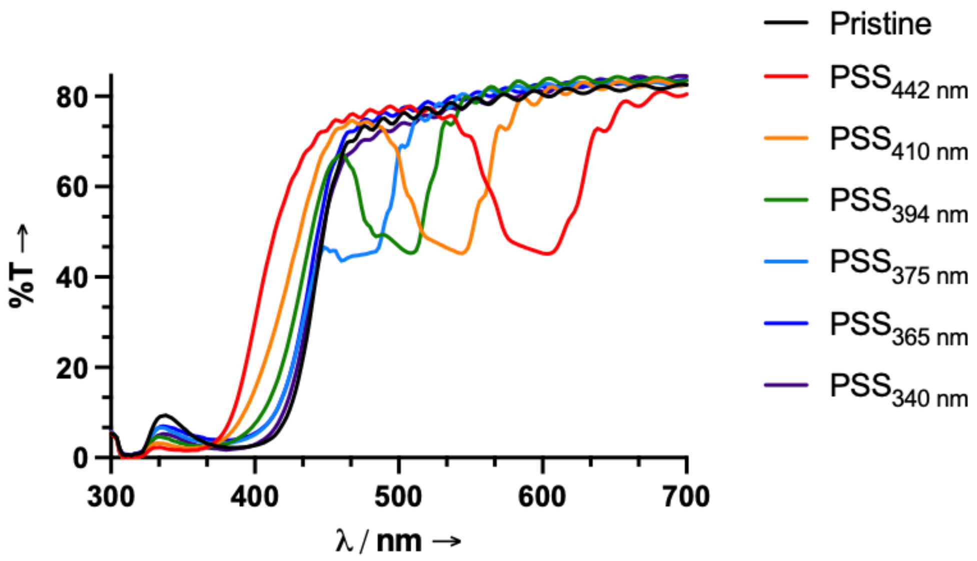

Figure S40: Reflectance modulation from a LC film containing compound (+)-(*M*)-**1** (3.4 mol%) and 5CB in Instec planar 3.0/5.0 cell as a function of PSS. Irradiation with 442 nm light results in the photoisomerization of *ZZZ* to *EEE* and a bathochromic shift in the transmitted light. Irradiation with 340 nm light results in the photoisomerization of *EEE* to *ZZZ* and a hypsochromic shift in the transmitted light.

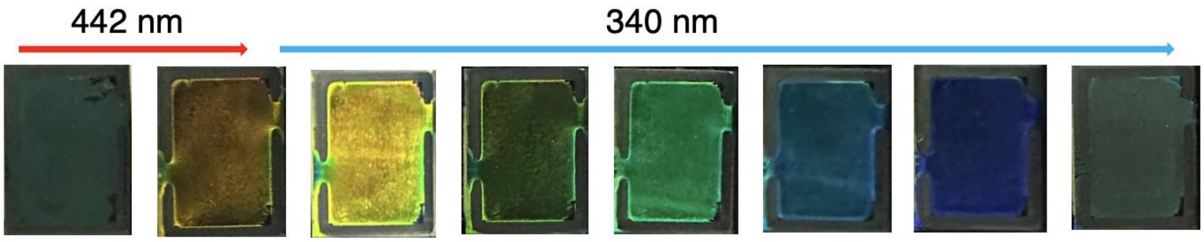

Figure S41: Reflectance modulation from a LC film containing compound (+)-(*M*)-**1** (3.4 mol%) and 5CB in Instec planar 3.0/5.0 cell upon irradiation with 442 and 340 nm light.

9. Co-Doping in Solution and Liquid Crystal

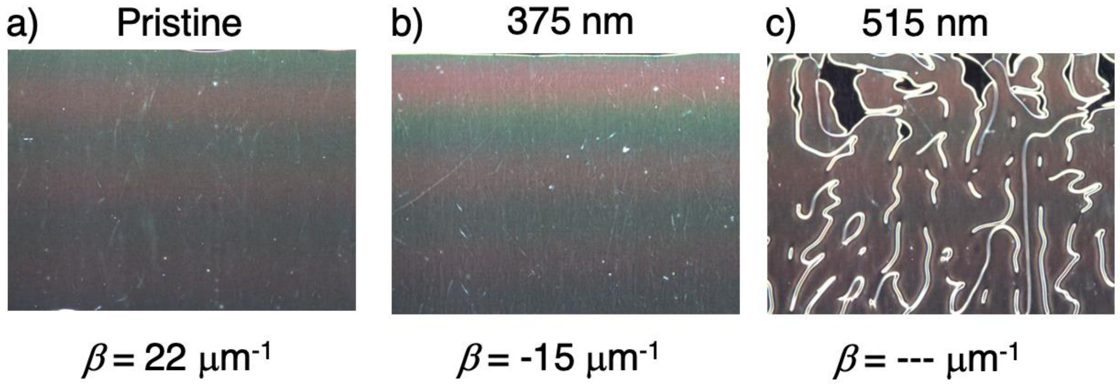

Figure S42: Photomicrographs of (-)-(*P*)-**1** and (+)-(*M*)-**2** (30:70, 0.23 mol%) in 5CB after sequentially irradiating (a) the pristine state with (b) 375, and (c) 515 nm light in a KCRK05 cell.

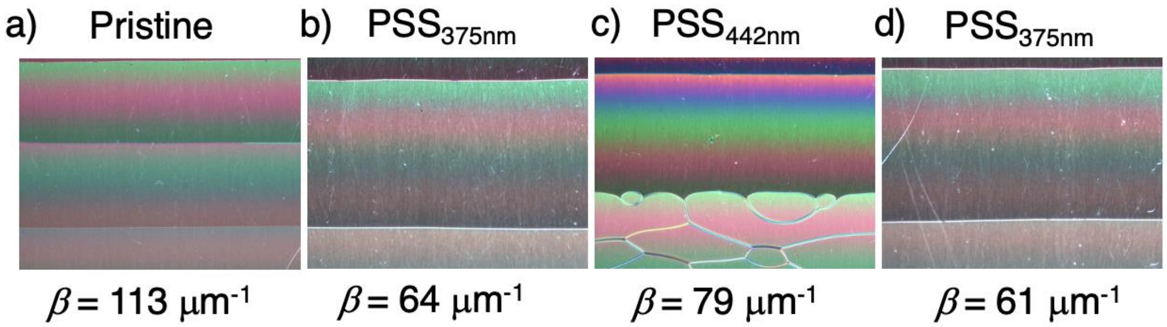

Figure S43: Photomicrographs of (+)-(*M*)-**1** and (+)-(*M*)-**2** (32:68, 0.12 mol%) in 5CB after sequentially irradiating (a) the pristine state with (b) 375, (c) 442, and (d) 375 nm light in a KCRK07 cell.

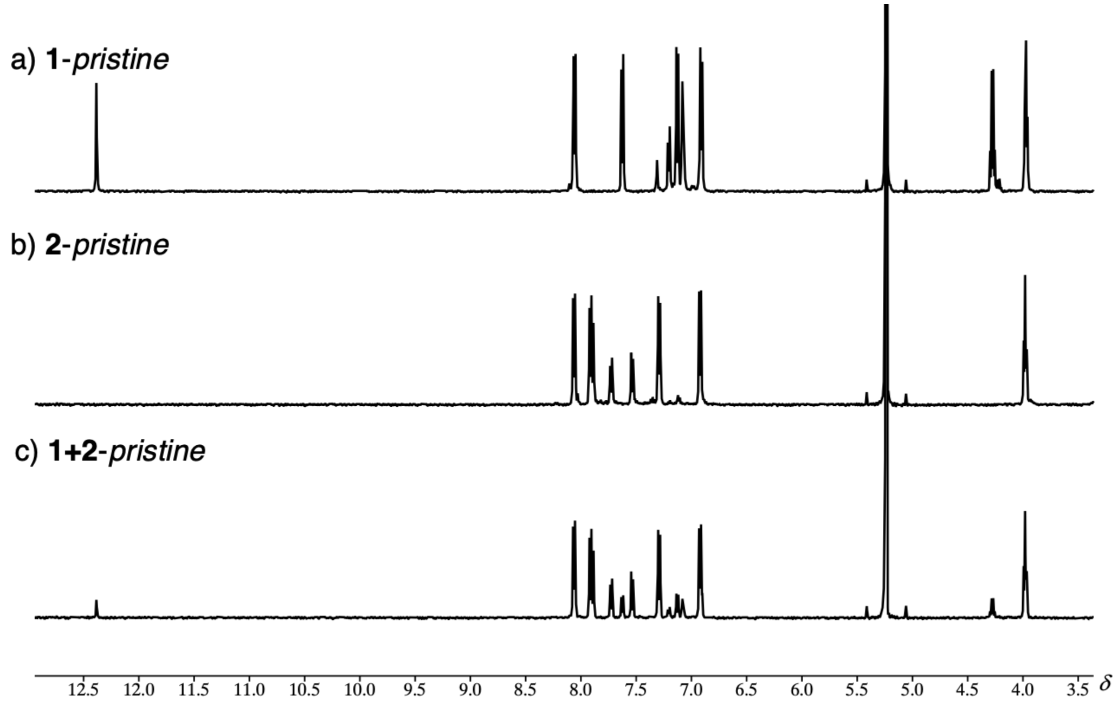

Figure S44: ^{1}H NMR spectra of the pristine sample (5 x 10^{-4} M) of (a) **1**, (b) **2**, and (c) **1** + **2** (1:4) in CD_2Cl_2 at 294K.

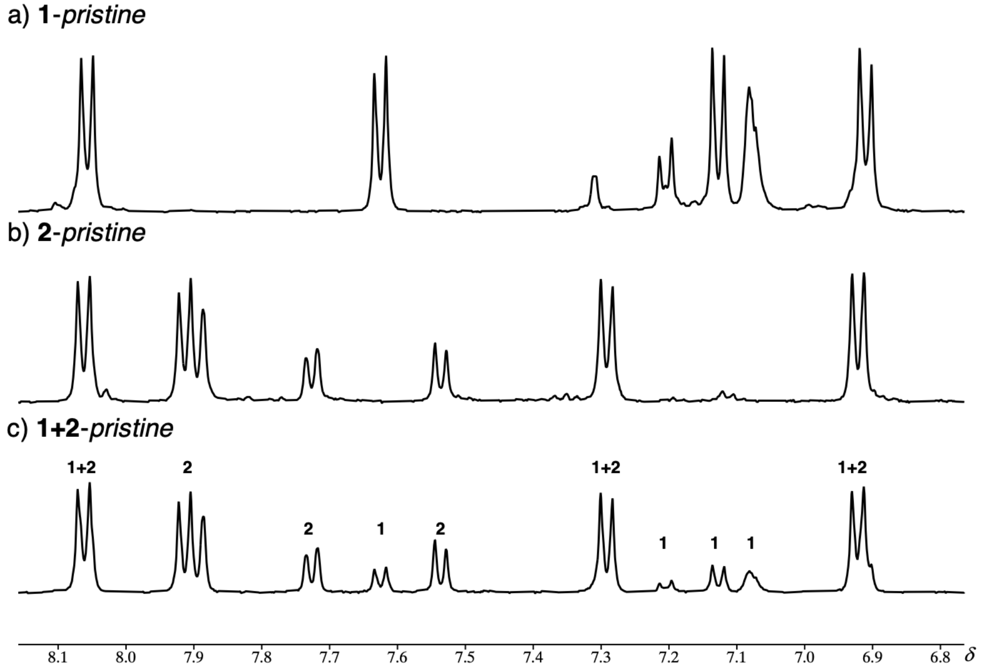

Figure S45: Zoom in of the ^{1}H NMR spectra of the pristine sample (5 x 10^{-4} M) of (a) **1**, (b) **2**, and (c) **1** + **2** (1:4) in CD_2Cl_2 at 294K.

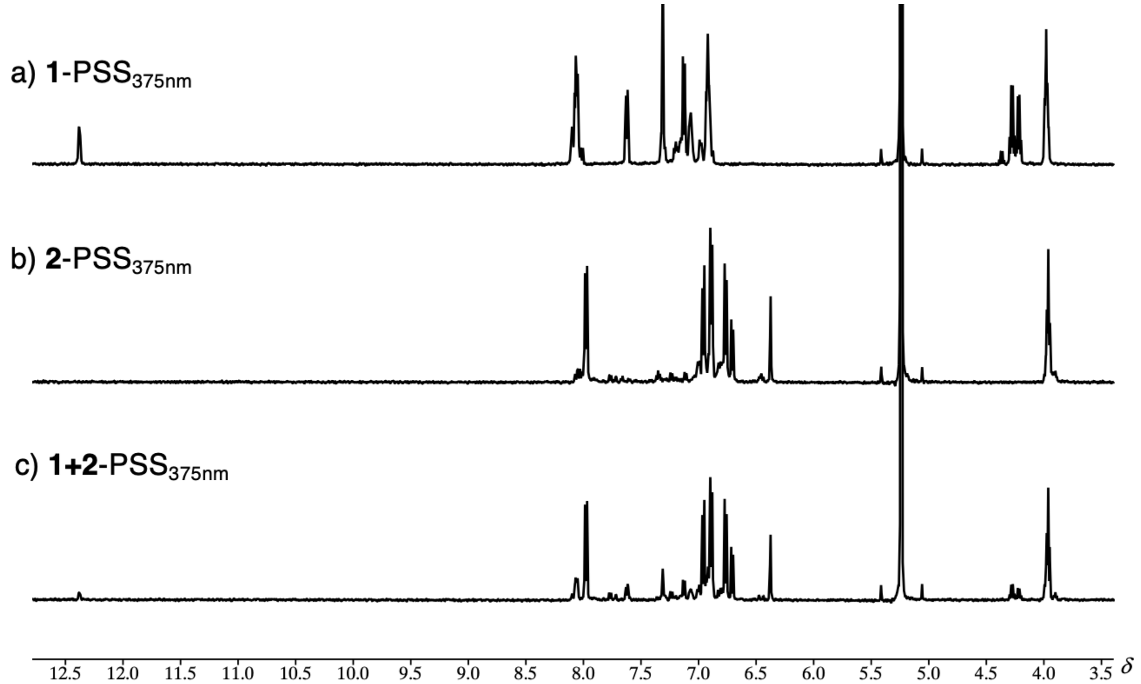

Figure S46: ^{1}H NMR spectra of the photostationary state (5 x 10^{-4} M) after irradiation with 375 nm light of (a) **1**, (b) **2**, and (c) **1** + **2** (1:4) in CD_2Cl_2 at 294K.

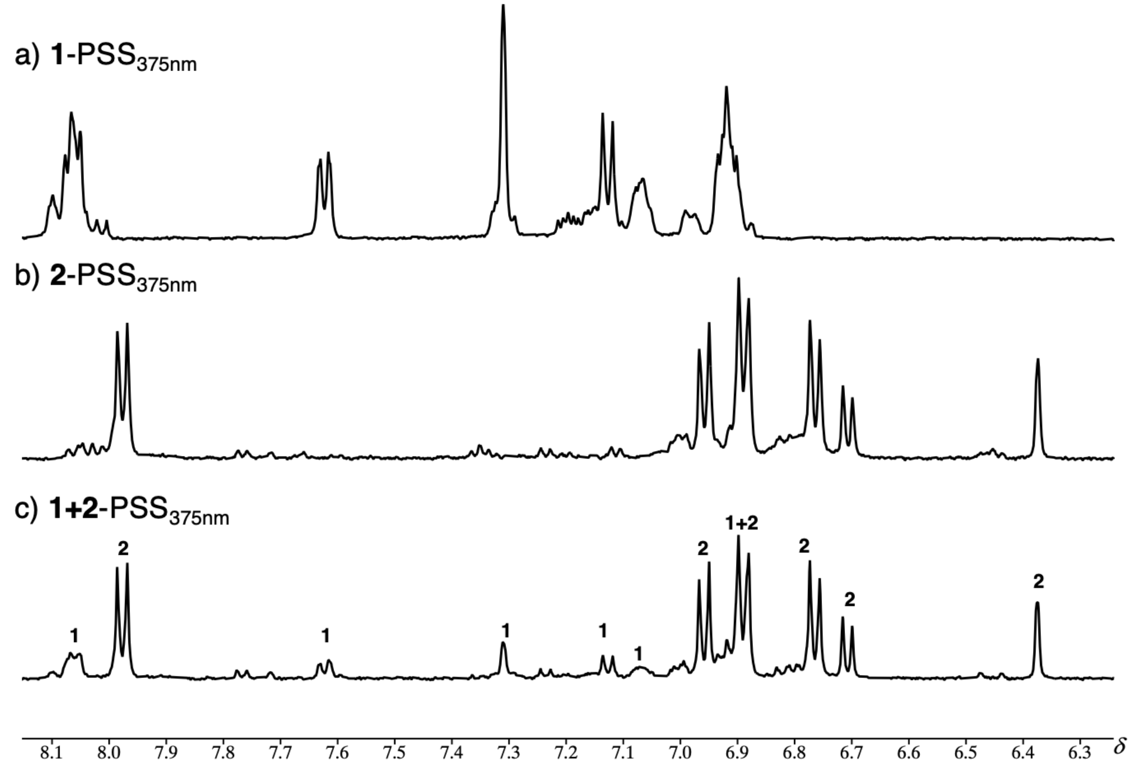

Figure S47: Zoom in of the ^{1}H NMR spectra of the photostationary state (5 x 10^{-4} M) after irradiation with 375 nm light of (a) **1**, (b) **2**, and (c) **1** + **2** (1:4) in CD_2Cl_2 at 294K.

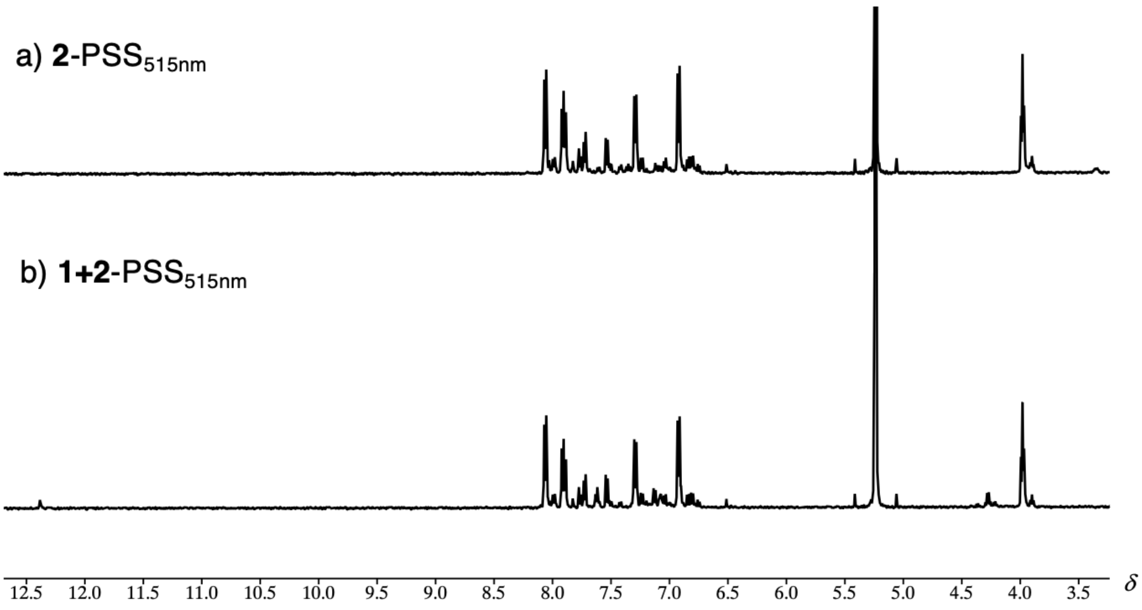

Figure S48: ^{1}H NMR spectra of the photostationary state (5 x 10^{-4} M) after irradiation with 515 nm light of (a) **2** and (b) **1** + **2** (1:4) in CD_2Cl_2 at 294K.

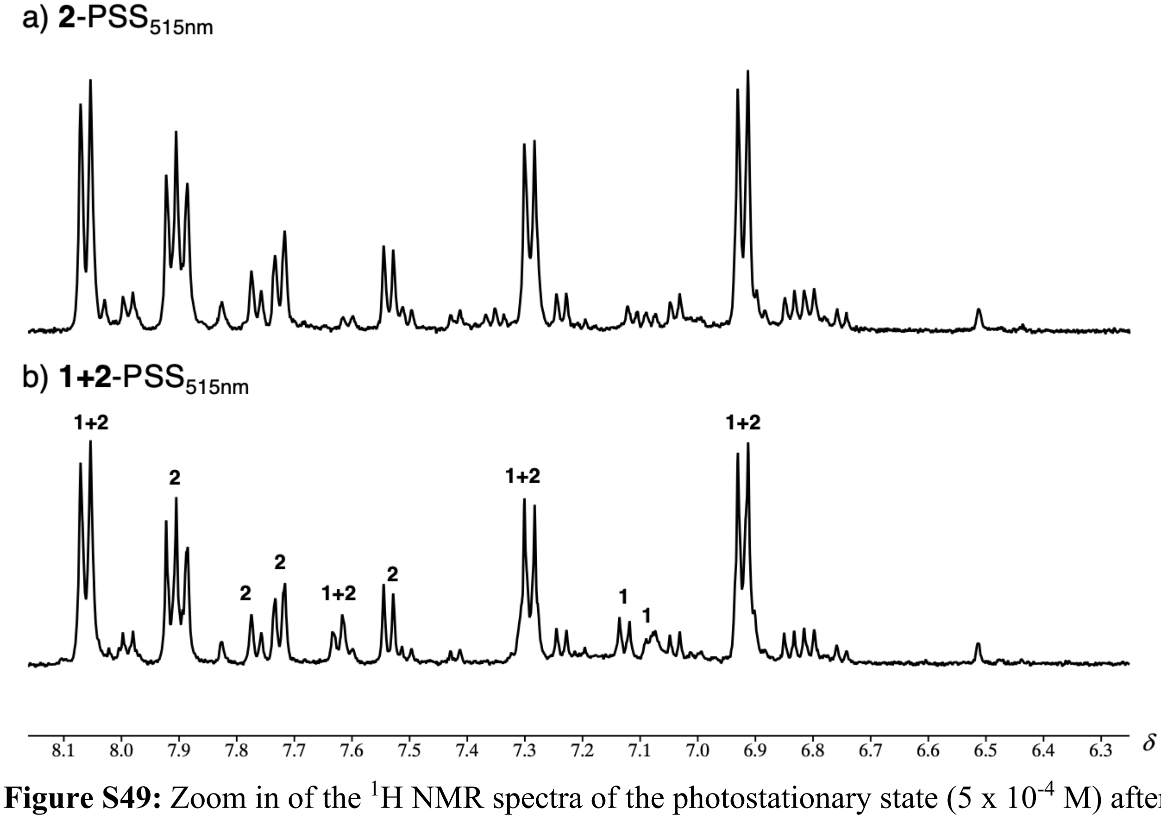

Figure S49: Zoom in of the ^{1}H NMR spectra of the photostationary state (5 x 10^{-4} M) after irradiation with 515 nm light of (a) **2** and (b) **1** + **2** (1:4) in CD_2Cl_2 at 294K.

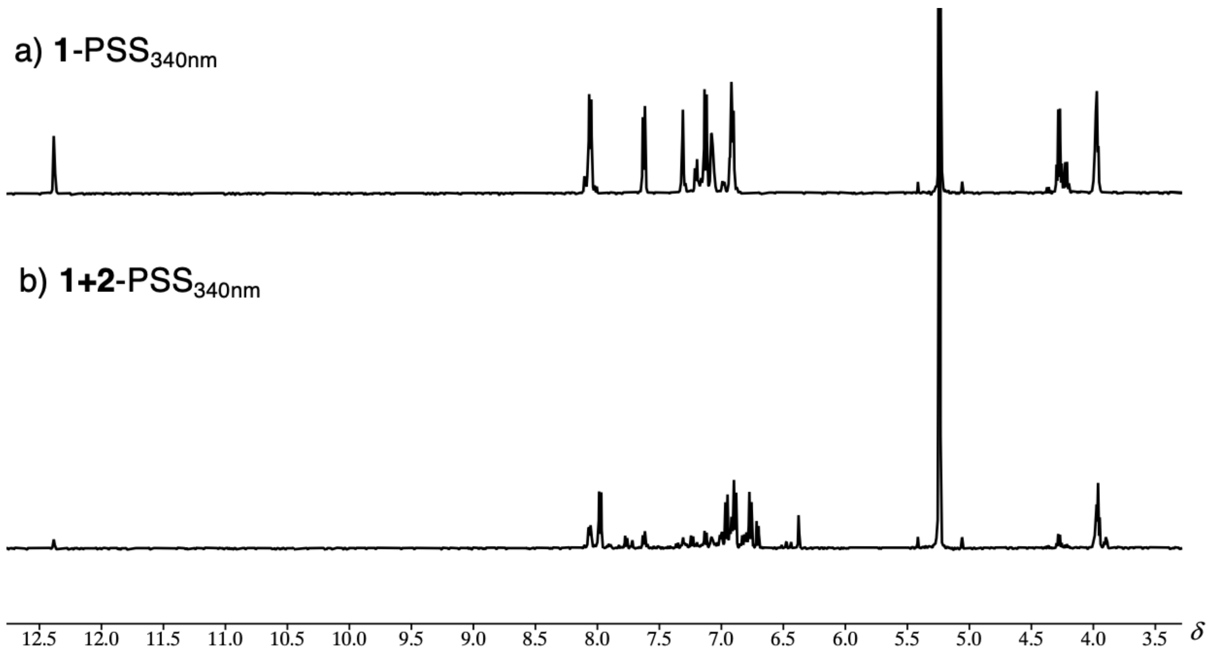

Figure S50: ^{1}H NMR spectra of the photostationary state (5 x 10^{-4} M) after irradiation with 340 nm light of (a) **1** and (b) **1** + **2** (1:4) in CD_2Cl_2 at 294K.

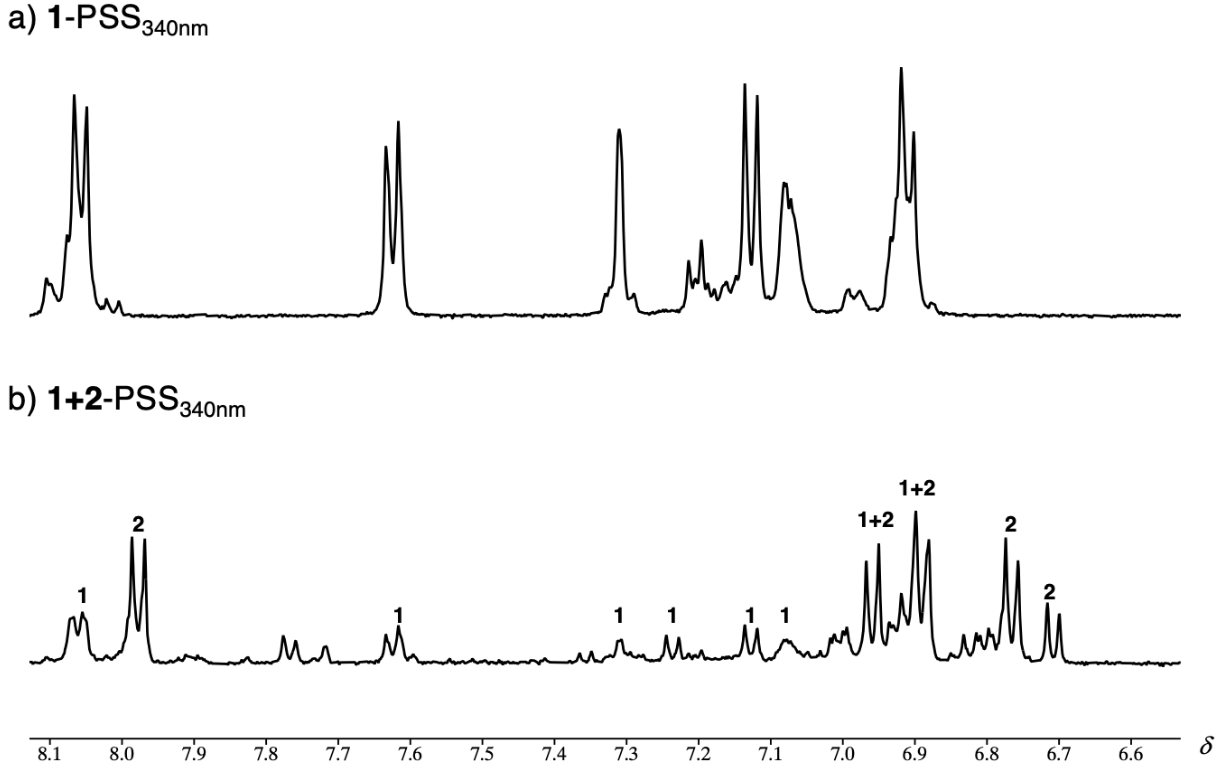

Figure S51: Zoom in of the ^{1}H NMR spectra of the photostationary state (5 x 10^{-4} M) after irradiation with 340 nm light of (a) **1** and (b) **1** + **2** (1:4) in CD_2Cl_2 at 294K.

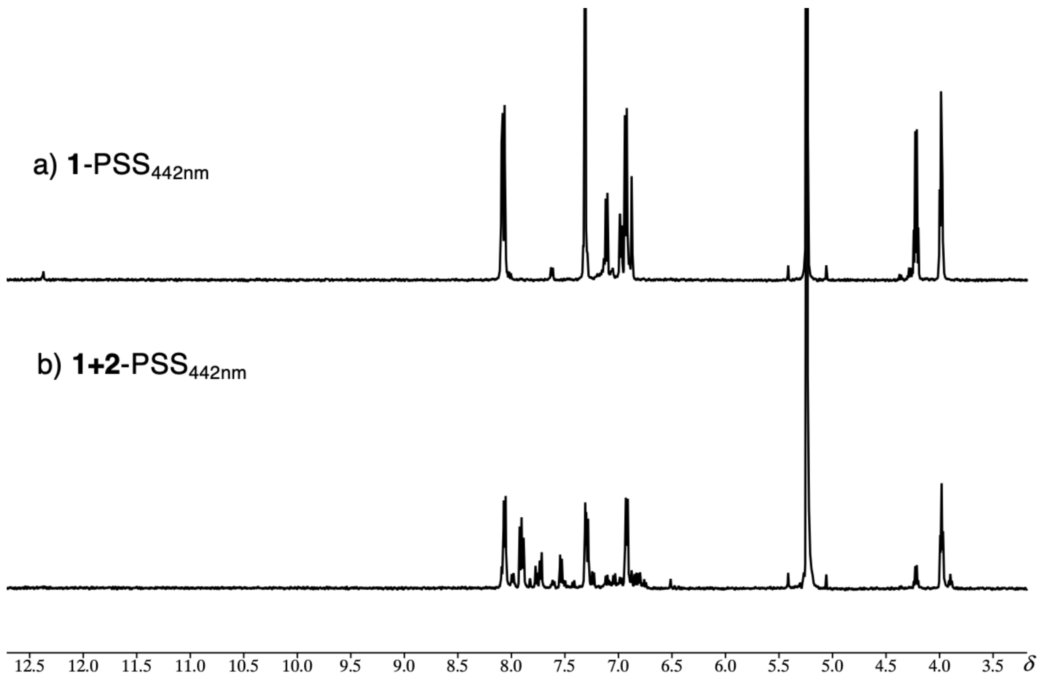

Figure S52: ^{1}H NMR spectra of the photostationary state (5 x 10^{-4} M) after irradiation with 442 nm light of (a) **1** and (b) **1** + **2** (1:4) in CD_2Cl_2 at 294K.

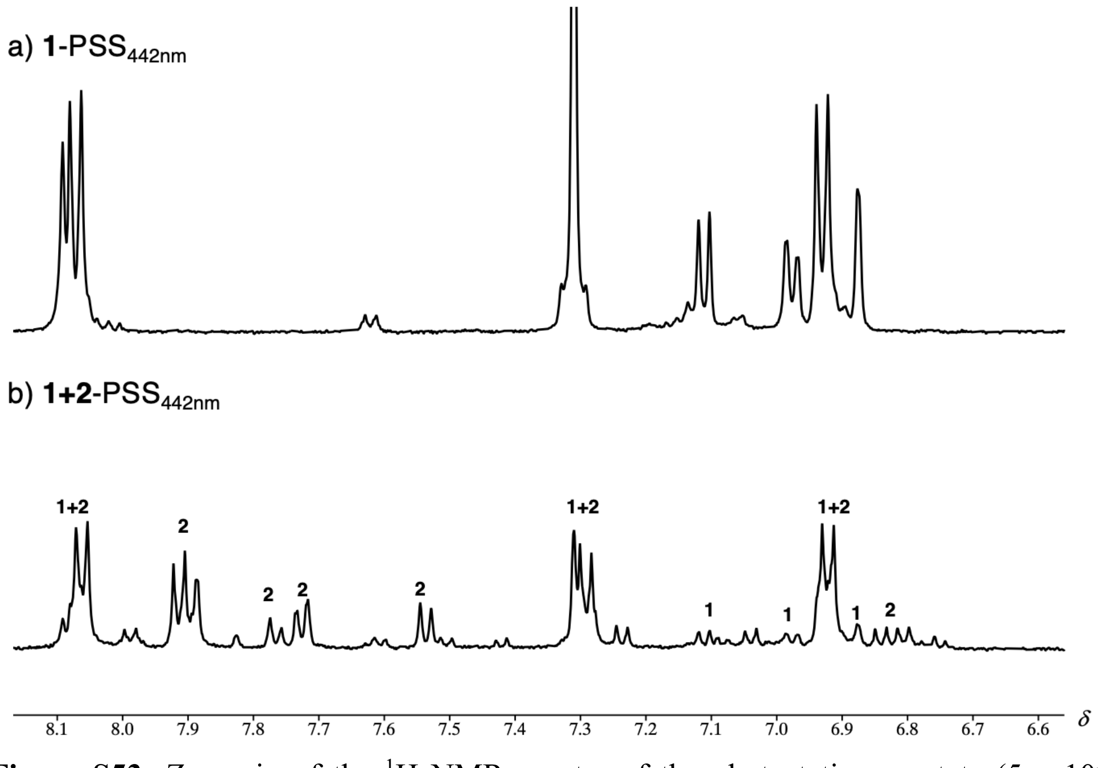

Figure S53: Zoom in of the ^{1}H NMR spectra of the photostationary state (5 x 10^{-4} M) after irradiation with 442 nm light of (a) **1** and (b) **1** + **2** (1:4) in CD_2Cl_2 at 294K.

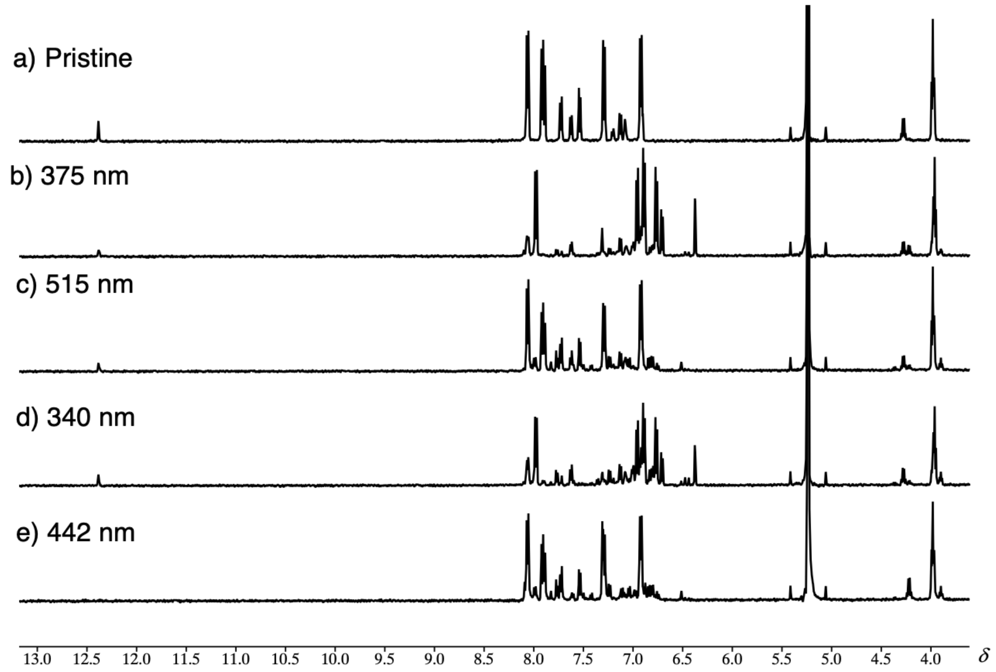

Figure S54: ^{1}H NMR spectra of (a) the pristine sample, (b) 375, (c) 515, (d) 340, and (e) 442 nm photostationary states of **1** + **2** (5 x 10^{-4} M) after sequential irradiation in CD_2Cl_2 at 294K.

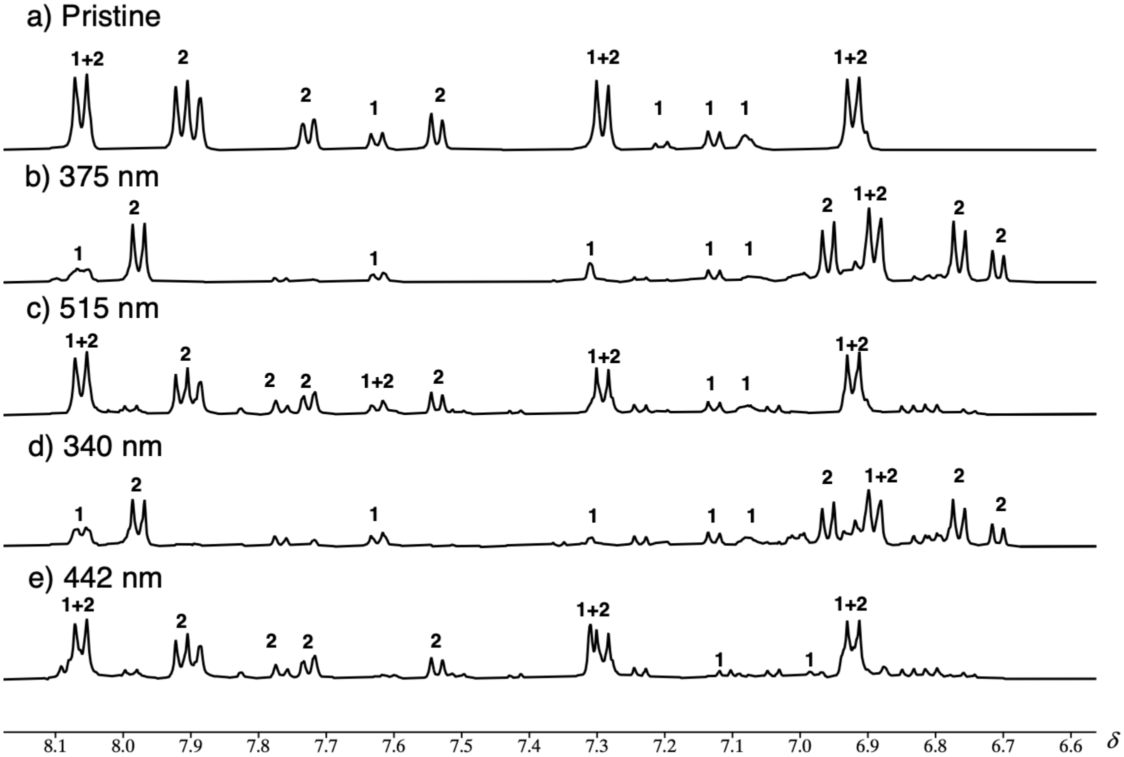

Figure S55: Zoom in of the ^{1}H NMR spectra of (a) the pristine sample, (b) 375, (c) 515, (d) 340, and (e) 442 nm photostationary states of **1** + **2** (5 x 10^{-4} M) after sequential irradiation in CD_2Cl_2 at 294K.

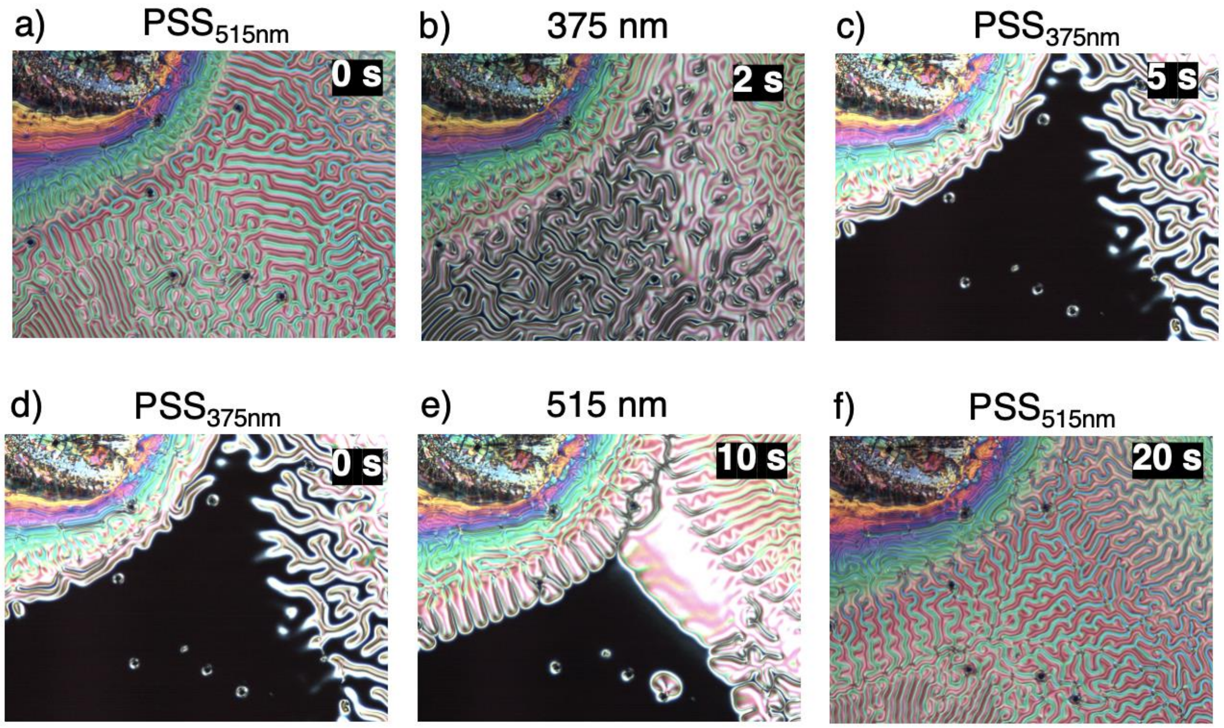

Figure S56: Cholesteric contact experiment using a reference left-handed cholesteric liquid crystal, cholesterol oleyl carbonate, and a mixture of (-)-(*P*)-**1** and (+)-(*M*)-**2** (30:70, 0.58 mol%) and 5CB in a 17 mm homeotropic cell. Irradiation with 375 nm light (a–c) results in helicity inversion after 5 seconds as evidenced by the emergence of a discontinuous region (black) at the interface between the LCs demonstrating a left-handed to right-handed transformation. Subsequent irradiation with 515 nm light (d–f) results in restoration of the starting helicity after 20 seconds as evidenced by the disappearance of the discontinuous region at the interface.

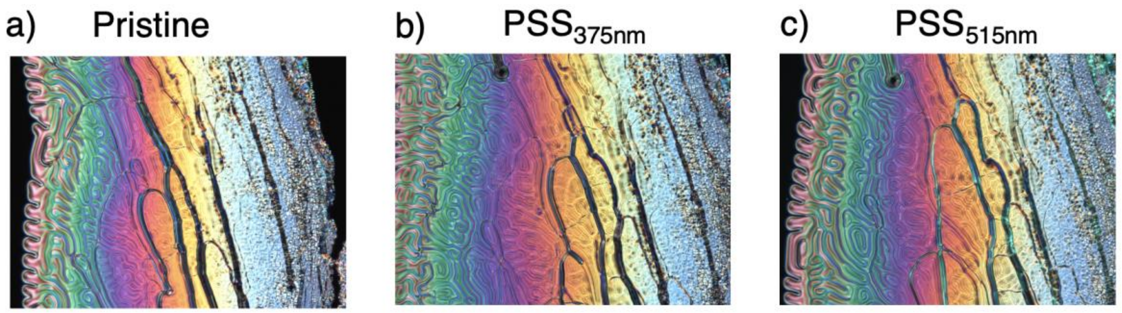

Figure S57: Cholesteric contact experiment using a reference left-handed cholesteric liquid crystal, cholesterol oleyl carbonate, and a mixture of (+)-(*M*)-**1** and (+)-(*M*)-**2** (32:68, 0.12 mol%) and 5CB in a 17 mm homeotropic cell. Sequential irradiation of the (a) pristine state with (b) 375 and (c) 515 nm light does not result in the inversion of helicity and the contact at the interface between the two LCs remains continuous.

10. References

(S1) Beaudoin, D.; Rominger, F.; Mastalerz, M. Synthesis and Chiral Resolution of C_3-Symmetric Tribenzotriquinacenes: Synthesis and Chiral Resolution of C_3-Symmetric Tribenzotriquinacenes. *Eur. J. Org. Chem.* **2016**, *2016*, 4470–4472.

(S2) Balamut, B.; Hughes, R. P.; Aprahamian, I. Tuning the Properties of Hydrazone/Isosorbide-Based Switchable Chiral Dopants. *J. Am. Chem. Soc.* **2024**, *146*, 24561–24569.

(S3) Qian, H.; Pramanik, S.; Aprahamian, I. Photochromic Hydrazone Switches with Extremely Long Thermal Half-Lives. *J. Am. Chem. Soc.* **2017**, *139*, 9140–9143.

(S4) He, Y.; Shangguan, Z.; Zhang, Z.; Xie, M.; Yu, C.; Li, T. Azobispyrazole Family as Photoswitches Combining (Near-) Quantitative Bidirectional Isomerization and Widely Tunable Thermal Half-Lives from Hours to Years *Angew. Chem. Int. Ed.* **2021**, *60*, 16539–16546.

(S5) The experimental Φ values are the observed Φ values measured from changes in the π-π* absorption bands of **1** and **2** as a function of time and are not considering the formation and isomerization of intermediate isomers.

(S6) Kuhn, H. J.; Braslavsky, S. E.; Schmidt, R. Chemical Actinometry (IUPAC Technical Report). *Pure and Applied Chemistry* **2004**, *76*, 2105–2146.

(S7) Connors, K. A. *Chemical Kinetics: The Study of Reaction Rates in Solution*, Nachdr.; Wiley-VCH: New York, NJ, 1990.

(S8) Moran, M. J.; Magrini, M.; Walba, D. M.; Aprahamian, I. Driving a Liquid Crystal Phase Transition Using a Photochromic Hydrazone. *J. Am. Chem. Soc.* **2018**, *140*, 13623–13627.

(S9) Bala, I.; Plank, J. T.; Balamut, B.; Henry, D.; Lippert, A. R.; Aprahamian, I. Multi-Stage and Multi-Colour Liquid Crystal Reflections Using a Chiral Triptycene Photoswitchable Dopant. *Nat. Chem.* **2024**, *16*, 2084–2090.
